# Supplementary material for: Rationale and study protocol of the Physical Activity and Dietary intervention in women with OVArian cancer (PADOVA) study: a randomised controlled trial to evaluate effectiveness of a tailored exercise and dietary intervention on body composition, physical function and fatigue in women with ovarian cancer undergoing chemotherapy
Source: BMJ Open. 2020 Nov 4;10(11):e036854. doi: 10.1136/bmjopen-2020-036854 (PMC7643503; doi:10.1136/bmjopen-2020-036854)
Supplement: Supplementary data [file bmjopen-2020-036854supp001.pdf]

Supplementary table A: Overview of Behavior Change Techniques (BCT's) used to promote health behavior change in the PADOVA dietary intervention

Theory	Construct	BCT[1]	Description of BCT
Social Cognitive Theory			
	Self-efficacy	Graded tasks	To promote self-efficacy, the dietitian will stimulate the participant to set easy to perform and achievable individual goals, and will promote gradually making individual goals more difficult until the recommendation is met.
	Outcome expectations	Comparative imagining of future outcomes	The dietitian will prompt or advise the imagining and comparing of future outcomes of changed versus unchanged behavior.
		Vicarious consequences	The dietitian will prompt observation of the consequences (including rewards and punishments) for others when they perform the behavior.
	Goal setting	Goal setting (outcomes of) behavior	Individual goals with regard to dietary intake/weight will be set by the participant in consultation with the dietitian.
	Sociostructural factors	Problem solving	The dietitian and patient discuss factors that could influence achieving each goal, as well as strategies to overcome possible barriers and/or strategies to increase facilitators to achieving each goal.
		Social support (practical)	The dietitian gives advice on finding social support (e.g. practical help from family or friends) for behavior change in order to reach individual goals.
		Habit formation	The dietitian will advise on rehearsal and repetition of the behavior in the same context repeatedly so that the context elicits the behavior.
		Avoidance/reducing exposure to cues for the behavior	The dietitian will advise on how to avoid exposure to specific social and contextual/physical cues for the behavior, including changing daily/weekly routines.
		Restructuring the physical environment	The dietitian will facilitate change or advise to change the physical environment in order to facilitate performance of the wanted behavior or create barriers to the unwanted behavior.
		Restructuring the social environment	The dietitian will facilitate change or advise to change the social environment in order to facilitate performance of the wanted behavior or create barriers to the unwanted behavior.
		Information about antecedents	The dietitian will provide information about antecedents (social, environmental situations or events, emotions, cognitions) that reliably predict performance of the behavior.
		Self-reward	The dietitian will prompt self-praise or self-reward if and only if there has been effort and/or progress in performing the behavior.
		Reduce negative emotions	The dietitian will advise on ways of reducing negative emotions to facilitate performance of the behavior (includes stress-management).
Motivational interviewing			
		Pros and cons	The dietitian will advise to identify and compare reasons for wanting (pros) and not wanting (cons) to change the behavior (includes decisional balance).
		Comparative imagining of future outcomes	The dietitian will prompt or advise the imagining and comparing of future outcomes of changed versus unchanged behavior.
		Social support (unspecified)	The dietitian will advise on and how to arrange social support (e.g., from friends, family, buddies) or non-contingent praise or reward for performance of the behavior. Includes encouragement and counselling when directed at the behavior.

Other			
		Credible source	Dietitian from hospital provides counselling.
		Feedback on behavior	The participant will receive feedback from the dietitian on diet quality, and on the extent to which they meet the World Cancer Research Fund (WCRF)/American Institute for Cancer Research (AICR) [2] recommendations and the protein-goals [3,4].
		Feedback on outcomes behavior	The participant will receive feedback from the dietitian on their weight, Body Mass Index, body composition.
		Information about health consequences	The dietitian will inform the participant about the influence of lifestyle-related factors on the occurrence of cancer and about the potential positive effects of increased physical activity and a healthy diet (including the effect of habitual protein consumption during exercise) throughout chemotherapy.
		Instruction on how to perform the behavior	The participant will receive information from the dietitian on the WCRF/AICR recommendations (leaflet).
		Adding objects to the environment; self-monitoring of behavior; self- monitoring of outcomes of behavior	The participant will receive a self-monitoring log from the dietitian in which they can log their weight and diet. They are encouraged to weekly log their weight, and to daily log their dietary intake, with flexibility to meet individual needs and preferences.
		Action planning	An action plan for each individual goal will be discussed by the participant and dietitian.
		Discrepancy between current behavior and goal	The dietitian will point out potential discrepancies between patients' current behavior and each goal during each subsequent session.
		Review behavior goals/review outcome goals	The self-monitoring logs will be discussed with the oncology dietitian during every counselling visit to be able to monitor progress. Each goal will be reviewed and may be modified if necessary. Also, new goals may be set.
		Social reward (positive reinforcement)	The dietitian will congratulate the patient in case of success.
		Verbal persuasion about capability	The dietitian will tell the person that they can successfully perform the wanted behavior, arguing against self-doubts and asserting that they can and will succeed.

REFERENCES

1. Michie S, Richardson M, Johnston M, et al. The behavior change technique taxonomy (v1) of 93 hierarchically clustered techniques: building an international consensus for the reporting of behavior change interventions. *Ann Behav Med* 2013;46(1):81-95. doi: 10.1007/s12160-013-9486-6 [published Online First: 2013/03/21]
2. World Cancer Research Fund / American Institute for Cancer Research. Diet, Nutrition, Physical Activity and Cancer: a Global Perspective. Continuous Update Project Expert report, 2018.
3. Nitenberg G, Raynard B. Nutritional support of the cancer patient: issues and dilemmas. *Crit Rev Oncol Hematol* 2000;34(3):137-68. [published Online First: 2000/06/06]
4. Arends J, Bachmann P, Baracos V, et al. ESPEN guidelines on nutrition in cancer patients. *Clin Nutr* 2016 doi: 10.1016/j.clnu.2016.07.015 [published Online First: 2016/09/18]

BMJ Publishing Group Limited (BMJ) disclaims all liability and responsibility arising from any reliance placed on this supplemental material which has been supplied by the author(s)
