# Supplementary material for: Rationale and study protocol of the Physical Activity and Dietary intervention in women with OVArian cancer (PADOVA) study: a randomised controlled trial to evaluate effectiveness of a tailored exercise and dietary intervention on body composition, physical function and fatigue in women with ovarian cancer undergoing chemotherapy
Source: BMJ Open. 2020 Nov 4;10(11):e036854. doi: 10.1136/bmjopen-2020-036854 (PMC7643503; doi:10.1136/bmjopen-2020-036854)
Supplement: Supplementary data [file bmjopen-2020-036854supp002.pdf]

Supplementary table B: Overview of the content of the PADOVA dietary counselling sessions

Counselling session	Content
First counselling session	- Introduction of dietitian and aim of dietary counselling sessions<br>- Anthropometric measures<br>o Current weight and weight history<br>o Height<br>o Body Mass Index<br>o Body composition<br>- Dietary assessment<br>o Nutrition-related illnesses or symptoms (e.g. reduced appetite, nausea, vomiting, gastrointestinal problems, chewing or swallowing difficulties)<br>o Relevant social factors (e.g. social support)<br>o Dietary analyses of current nutritional intake<br>o Current exercise and physical activity level<br>- Assessment of energy [1,2] and protein [3,4] requirements using multiple formulas<br>- Dietetic diagnosis (synthesized information from anthropometric measures and dietary assessment)<br>- Provide feedback on patients' weight, body composition and dietary intake<br>- Providing information about the influence of lifestyle and body weight related factors on the occurrence of cancer and about the potential positive effects of increased physical activity and a healthy diet (including the effects of habitual protein consumption during exercise) throughout chemotherapy.<br>- Set individual goals and action plans to achieve goals (depending on current nutritional status)<br>- Discussion of factors that could influence achieving each goal, as well as strategies to overcome possible barriers and/or strategies to increase facilitators<br>- Hand out self-monitoring logs in which patients can log their weight, dietary intake and/or physical activity.
Second – fifth counselling session	- Anthropometric measures<br>o Current weight and Body Mass Index<br>o Body composition (every other counselling session)<br>- Dietary assessment (if changed)<br>- Assessment of energy and protein requirements (if changed)<br>- (Revision of) dietetic diagnosis<br>- Discussion of filled in self-monitoring logs<br>- Discussion of potential discrepancies between current behavior and each goal<br>- Review and if necessary modification of goals and action plans
Last counselling session	- Same content as second to fifth counselling session<br>- Discussion and encouragement of self-regulation strategies to be able to maintain adherence to the World Cancer Research Fund/American Institute for Cancer Research recommendations after the end of the intervention [5]

REFERENCES

1. Roza AM, Shizgal HM. The Harris Benedict equation reevaluated: resting energy requirements and the body cell mass. *Am J Clin Nutr* 1984;40(1):168-82. doi: 10.1093/ajcn/40.1.168 [published Online First: 1984/07/01]
2. Energy and protein requirements. Report of a joint FAO/WHO/UNU Expert Consultation. *World Health Organ Tech Rep Ser* 1985;724:1-206. [published Online First: 1985/01/01]
3. Nitenberg G, Raynard B. Nutritional support of the cancer patient: issues and dilemmas. *Crit Rev Oncol Hematol* 2000;34(3):137-68. [published Online First: 2000/06/06]
4. Arends J, Bachmann P, Baracos V, et al. ESPEN guidelines on nutrition in cancer patients. *Clin Nutr* 2016 doi: 10.1016/j.clnu.2016.07.015 [published Online First: 2016/09/18]
5. World Cancer Research Fund / American Institute for Cancer Research. Diet, Nutrition, Physical Activity and Cancer: a Global Perspective. Continuous Update Project Expert report, 2018.
