# Supplementary material for: Rationale and study protocol of the Physical Activity and Dietary intervention in women with OVArian cancer (PADOVA) study: a randomised controlled trial to evaluate effectiveness of a tailored exercise and dietary intervention on body composition, physical function and fatigue in women with ovarian cancer undergoing chemotherapy
Source: BMJ Open. 2020 Nov 4;10(11):e036854. doi: 10.1136/bmjopen-2020-036854 (PMC7643503; doi:10.1136/bmjopen-2020-036854)
Supplement: Author's manuscript [file bmjopen-2020-036854.draft_revisions.pdf]

BMJ Open

BMJ Open is committed to open peer review. As part of this commitment we make the peer review history of every article we publish publicly available.

When an article is published we post the peer reviewers' comments and the authors' responses online. We also post the versions of the paper that were used during peer review. These are the versions that the peer review comments apply to.

The versions of the paper that follow are the versions that were submitted during the peer review process. They are not the versions of record or the final published versions. They should not be cited or distributed as the published version of this manuscript.

BMJ Open is an open access journal and the full, final, typeset and author-corrected version of record of the manuscript is available on our site with no access controls, subscription charges or pay-per-view fees (http://bmjopen.bmj.com).

If you have any questions on BMJ Open's open peer review process please email info.bmjopen@bmj.com

BMJ Open

Rationale and design of the Physical Activity and Dietary intervention in women with OVArian cancer (PADOVA) study: A randomised controlled trial to evaluate effectiveness of a tailored exercise and dietary intervention on body composition, physical function and fatigue in patients with ovarian cancer undergoing chemotherapy

Journal:	*BMJ Open*
Manuscript ID	bmjopen-2020-036854
Article Type:	Protocol
Date Submitted by the Author:	07-Jan-2020
Complete List of Authors:	Stelten, Stephanie; Amsterdam UMC - Locatie VUMC, Epidemiology and Biostatistics<br>Hoedjes, Meeke ; Tilburg University, Medical and Clinical Psychology<br>Kenter, Gemma; Amsterdam UMC - Locatie AMC, Obstetrics and Gynaecology; Amsterdam UMC - Locatie VUMC, Obstetrics and Gynaecology<br>Kampman, Ellen; Wageningen University and Research, Division of Human Nutrition and Health<br>Huijsmans, Rosalie; Amsterdam UMC - Locatie VUMC, Rehabilitation Medicine<br>van Lonkhuijzen, Luc; Amsterdam UMC - Locatie AMC, Obstetrics and Gynaecology<br>Buffart, LM; Amsterdam UMC - Locatie VUMC, Epidemiology & Biostatistics and Medical Oncology
Keywords:	Nutritional support < ONCOLOGY, Gynaecological oncology < ONCOLOGY, NUTRITION & DIETETICS, SPORTS MEDICINE, REHABILITATION MEDICINE, Epidemiology < ONCOLOGY

SCHOLARONE™
Manuscripts

I, the Submitting Author has the right to grant and does grant on behalf of all authors of the Work (as defined in the below author licence), an exclusive licence and/or a non-exclusive licence for contributions from authors who are: i) UK Crown employees; ii) where BMJ has agreed a CC-BY licence shall apply, and/or iii) in accordance with the terms applicable for US Federal Government officers or employees acting as part of their official duties; on a worldwide, perpetual, irrevocable, royalty-free basis to BMJ Publishing Group Ltd ("BMJ") its licensees and where the relevant Journal is co-owned by BMJ to the co-owners of the Journal, to publish the Work in this journal and any other BMJ products and to exploit all rights, as set out in our licence.

The Submitting Author accepts and understands that any supply made under these terms is made by BMJ to the Submitting Author unless you are acting as an employee on behalf of your employer or a postgraduate student of an affiliated institution which is paying any applicable article publishing charge ("APC") for Open Access articles. Where the Submitting Author wishes to make the Work available on an Open Access basis (and intends to pay the relevant APC), the terms of reuse of such Open Access shall be governed by a Creative Commons licence – details of these licences and which Creative Commons licence will apply to this Work are set out in our licence referred to above.

Other than as permitted in any relevant BMJ Author's Self Archiving Policies, I confirm this Work has not been accepted for publication elsewhere, is not being considered for publication elsewhere and does not duplicate material already published. I confirm all authors consent to publication of this Work and authorise the granting of this licence.

For peer review only

Rationale and design of the Physical Activity and Dietary intervention in women with OVArian cancer (PADOVA) study: A randomised controlled trial to evaluate effectiveness of a tailored exercise and dietary intervention on body composition, physical function and fatigue in patients with ovarian cancer undergoing chemotherapy

S. Stelten[1], M. Hoedjes[2], G.G. Kenter[3,4,5], E. Kampman[6], R.J. Huijsmans[7], L.R.C.W. van Lonkhuijzen[4], L.M. Buffart[1,8]

Correspondence

Laurien M. Buffart

De Boelelaan 1089a

1081 HV Amsterdam, the Netherlands

E-mail address: l.buffart@amsterdamumc.nl

Tel: +31 20 444 9931

Authors affiliations

[1]Amsterdam UMC, Vrije Universiteit Amsterdam, Department of Epidemiology and Biostatistics, Amsterdam Public Health research institute, Cancer Center Amsterdam, de Boelelaan 1089a, 1081 HV Amsterdam, Netherlands

[2]Tilburg University, Center of Research on Psychology in Somatic diseases, Department of Medical and Clinical Psychology, Warandelaan 2, 5037 AB Tilburg, Netherlands

[3]Amsterdam UMC, Vrije Universiteit Amsterdam, Department of Obstetrics and Gynaecology, Cancer Center Amsterdam, Center for Gynaecologic Oncology Amsterdam (CGOA), de Boelelaan 1117, 1081 HV Amsterdam, Netherlands

[4]Amsterdam UMC, Univ(ersity) of Amsterdam, Department of Obstetrics and Gynaecology, Center for Gynaecologic Oncology Amsterdam (CGOA), Meibergdreef 9, 1105 AZ Amsterdam, Netherlands

[5]The Netherlands Cancer Institute – Antoni van Leeuwenhoek Hospital, Department of Gynaecology, Center for Gynaecologic Oncology Amsterdam (CGOA), Plesmanlaan 121, 1066 CX Amsterdam, Netherlands

[6]Wageningen University and Research, Division of Human Nutrition and Health, P.O. Box 17, 6700 AA Wageningen, Netherlands

[7]Amsterdam UMC, Vrije Universiteit van Amsterdam, Department of Rehabilitation Medicine, de

For peer review only - http://bmjopen.bmj.com/site/about/guidelines.xhtml

Boelelaan 1117, 1081HV Amsterdam, Netherlands

[8]Amsterdam UMC, Vrije Universiteit Amsterdam, Department of Medical Oncology, Cancer Center Amsterdam, de Boelelaan 1117, 1081 HV Amsterdam, Netherlands

Word count: 3785 (excluding title page, abstract, references, figures and tables)

For peer review only

For peer review only - http://bmjopen.bmj.com/site/about/guidelines.xhtml

ABSTRACT

Introduction: As a consequence of ovarian cancer and its treatment, many women with ovarian cancer have to deal with fatigue, reduced physical function and loss of weight and/or muscle mass, compromising quality of life. Exercise and dietary interventions can positively influence body composition, physical fitness and function and fatigue in patients with cancer. However, there is no data from randomised controlled trials on the effectiveness of exercise and dietary interventions in patients with ovarian cancer. Due to a complex disease trajectory, a relatively poor survival and distinct disease- and treatment-induced side effects, it is unclear whether exercise and dietary interventions that were shown feasible and effective in other types of cancer produce comparable results in patients with ovarian cancer. The aim of this paper is to present the design of the multicenter randomised controlled Physical Activity and Dietary intervention in OVArian cancer (PADOVA) trial and to describe how the exercise and dietary intervention is tailored to specific comorbidities, and disease- and treatment induced adverse effects in patients with ovarian cancer.

Methods and analysis: Adult women with primary epithelial ovarian cancer who are scheduled to undergo first-line (neo)adjuvant chemotherapy are randomly allocated to a combined exercise and dietary intervention or a usual care control group during chemotherapy. Primary outcomes are body composition, physical function and fatigue. Outcome measures will be assessed before the start of chemotherapy, three weeks after completion of chemotherapy and 12 weeks later. The exercise and dietary intervention was tailored to ovarian cancer specific comorbidities and adverse effects of ovarian cancer and its treatment following the i3-S strategy.

Ethics and dissemination: This study has been approved by the medical ethical committee of the Amsterdam UMC (reference: 018). Results of the study will be published in international peer-reviewed journals.

Trial registration number: Netherlands Trial register (NTR6300)

Strengths and limitations of this study

- This is a randomised controlled trial in a relatively large group of women with ovarian cancer.
- Systematic development of the exercise and dietary intervention will improve compliance to the intervention and prevent drop out.

For peer review only

For peer review only - http://bmjopen.bmj.com/site/about/guidelines.xhtml

- This study will significantly contribute to the scientific evidence on the benefits of exercise and dietary support in an understudied group of patients.
- This study offers a combined exercise and dietary intervention and therefore the effects of an exercise or dietary intervention alone cannot be disentangled.
- There long-term effectiveness of the intervention may be diluted because the usual care group will be offered physical activity and dietary counselling after completion of chemotherapy treatment.

INTRODUCTION

Ovarian cancer is the seventh most common type of cancer worldwide, with 239.000 new cases annually.[1] Ovarian cancer is often diagnosed at an advanced International Federation of Gynaecology and Obstetrics (FIGO) stage resulting in a poor overall prognosis.[2] The overall 5-year survival rate for ovarian cancer is 30-40%, but ranges from 92% in patients with FIGO stage I at diagnosis to 29% in patients with FIGO stage IV.[2]

The majority (90%) of malignant ovarian tumors are of epithelial origin.[2] Standard care for epithelial ovarian cancer includes cytoreductive surgery and platinum- and taxane-based (neo)adjuvant chemotherapy.[3,4] As a consequence of ovarian cancer and its treatment, many women have physical and/or psychosocial problems such as fatigue and reduced physical function, compromising quality of life.[5-14] Additionally, previous studies reported that half of the patients suffer from sarcopenia (i.e. loss of skeletal muscle mass) at diagnosis and that the prevalence increased during neoadjuvant chemotherapy.[9,10] Other studies reported that 24-57% were overweight and 10-35% obese.[9,10,15-19] Independent from Body Mass Index (BMI), risk of malnutrition in patients with ovarian cancer is high. Studies reported that before start of and during treatment of ovarian cancer 44-67% of patients are malnourished.[20,21] Observational studies in women with ovarian cancer found that sarcopenia, overweight and obesity at diagnosis, as well as loss of body weight and muscle mass during treatment, and underweight after treatment were associated with a lower survival rate.[9-11,15-19,22] Observational studies among patients with cancer, not including ovarian cancer, have shown that higher levels of physical activity or physical fitness are associated with better survival.[23,24] Therefore, it may be important to prevent weight gain or involuntary weight loss, and maintain physical fitness and muscle mass during treatment.

For peer review only - http://bmjopen.bmj.com/site/about/guidelines.xhtml

For peer review only

Exercise and dietary interventions are both non-pharmacological interventions that can positively influence body composition, physical fitness and function and reduce fatigue in patients with cancer.[25-29] A meta-analysis of intervention studies among the general population[30] as well as the International Society of Sports Nutrition[31] highlight that a combined exercise and dietary intervention is more effective for changing body composition than an exercise or dietary intervention alone. Most previous studies in patients with cancer were conducted in patients with breast cancer. It is unclear whether the effects of exercise and dietary interventions found in patients with breast cancer can be generalized to women with ovarian cancer. Compared with breast cancer, ovarian cancer is often detected in a more advanced stage[32] and in older women. Ovarian cancer also has a substantially different treatment trajectory, i.e. different type of chemotherapy and other adjuvant therapy regimens. A few previous pilot studies have indicated that low-to-moderate intensity exercise interventions[33,34] or a combined exercise and dietary intervention[35] during chemotherapy are feasible in women with ovarian cancer. To date, large randomised controlled trials evaluating the effect of an exercise and dietary intervention during treatment in patients with ovarian cancer are lacking. Therefore, the Physical Activity and Dietary intervention in OVArian cancer (PADOVA) study was initiated. The PADOVA study aims to evaluate the effectiveness of a combined moderate-to-high intensity exercise and dietary intervention during chemotherapy on body composition, physical function, and fatigue as primary outcomes, compared to an usual care control group in women undergoing chemotherapy for ovarian cancer. Secondary outcomes are physical activity and fitness, dietary intake, BMI, patients reported outcomes and treatment toxicity and completion rates. The secondary aim is to conduct an extensive process evaluation to examine how and why the intervention is (in)effective.

To optimize intervention feasibility and study retention rates, we aim to offer exercise and dietary interventions that are specifically tailored to the comorbidities, disease- and treatment induced adverse effects that individual patients with ovarian cancer may face. This paper presents the design of the multicenter randomised controlled PADOVA trial, and describes how the combined exercise and dietary intervention can be tailored specifically to patients with ovarian cancer.

METHODS AND ANALYSIS

The PADOVA study is a multicenter, single blind, randomised controlled trial. This study is funded by Dutch Cancer Society (VU 2015-7950), sponsored by Amsterdam UMC and approved by the medical

For peer review only - http://bmjopen.bmj.com/site/about/guidelines.xhtml

ethical committee of Amsterdam UMC. Patient inclusion and data collection has started in February 2018 and is currently ongoing.

Participants

The study aims to include 122 adult (aged ≥ 18 years) women who are scheduled for (neo)adjuvant first-line chemotherapy treatment for primary epithelial ovarian cancer. Patients are excluded from this study when they have had a prior cancer diagnosis within 5 years, are not able to perform basic activities of daily living, have a contraindication for exercise (e.g. heart failure), have a cognitive disorder or severe emotional instability (e.g. schizophrenia, Alzheimer), are unable to read and/or write Dutch or have a life expectancy of less than 3 months. Written informed consent is obtained from all patients prior to participation.

Recruitment and randomisation

Patients are recruited from the Center of Gynaecologic Oncology Amsterdam, which is a collaboration of all gynaecological oncologists in Amsterdam from Amsterdam UMC and the Netherlands Cancer Institute – Antoni van Leeuwenhoek. After baseline measurement, participants are stratified by FIGO stage (low (I/II) versus high (III/IV stage) and chemotherapy regimen (primary surgery followed by chemotherapy versus neoadjuvant chemotherapy followed by interval debulking and adjuvant chemotherapy) and randomly allocated to either a combined exercise and dietary intervention or a usual care group. An independent researcher performs the randomisation by using a table of random numbers in blocks of four generated by an independent statistician. Allocation sequence was concealed from the research and clinical staff. After randomisation, patients in both the intervention and the control group will receive a brochure on physical activity, diet and body weight recommendations for cancer survivors[36]. Patients who do not wish to participate in the study are invited to complete a single questionnaire examining relevant characteristics and reasons for declining participation to verify representatives of the study population. See Figure 1 for an overview of the study design and procedures.

Development and description of the combined exercise and dietary intervention

The aims of the exercise and dietary intervention are to maintain physical fitness and function, to prevent

For peer review only - http://bmjopen.bmj.com/site/about/guidelines.xhtml

the loss of lean body mass and to maintain a healthy body weight during (neo)adjuvant chemotherapy. The intervention starts at the first cycle of chemotherapy and continues until three weeks after the last cycle (duration of ±18 weeks).

For optimal intervention feasibility and study retention rates it is important to offer an exercise and dietary intervention in the PADOVA study which is specifically tailored to patients with ovarian cancer. The exercise intervention was based on the exercise intervention that has previously been shown to effectively maintain physical fitness, limit fatigue and enhance quality of life during chemotherapy in patients with breast cancer.[28] The dietary intervention was based on Dutch and international guidelines on general nutritional support for patients with cancer and nutritional support for malnourished patients or patients with ovarian cancer.[37-41] These interventions were tailored via the I3-s strategy to ovarian cancer specific comorbidities, adverse effects of ovarian cancer (e.g. ascites) and its surgical and chemotherapy treatment. The I3-S strategy was introduced in 2015 by Dekker et al.[42] to develop comorbidity-related adaptations to exercise therapy, and has previously been used to tailor exercise interventions to potential comorbidities and adverse effects of breast cancer treatment[43] and patients with knee osteoarthritis.[44]

The I3-S strategy consists of four steps, via which relevant information on the specific disease is collected. In the first step, information on comorbidities that occur in patients with ovarian cancer was gathered. All registered comorbidities of patients (n=109) who were treated for ovarian cancer in 2016 in the Amsterdam UMC were collected from patients records. Comorbidities were categorized according to International Classification of Diseases, 10th revision[45] (see Table 1 for an overview of all comorbidities).

For peer review only

For peer review only - http://bmjopen.bmj.com/site/about/guidelines.xhtml

Table 1: Comorbidities in patients with ovarian cancer (N=109)

**Comorbidity**	**%**
Hypertensive diseases (i.e. hypertension)	28
Ischaemic heart diseases (i.e. angina pectoris, myocardial infarction (>6 months), percutaneous transluminal coronary angioplasty, coronary artery bypass grafting)	6
Other forms of heart disease (i.e. atrial fibrillation and flutter, other cardiac arrhythmias, heart failure, other forms of heart disease not specified)	9
Cerebrovascular diseases (i.e. stroke, not specified as haemorrhage or infarction)	4
Diseases of arteries, arterioles and capillaries (i.e. aortic aneurysm, peripheral vascular diseases)	3
Diseases of veins, lymphatic vessels and lymph nodes, not elsewhere classified (i.e. phlebitis and thrombophlebitis)	1
Chronic lower respiratory diseases (i.e. chronic obstructive pulmonary disease, asthma)	16
Diabetes Mellitus (unspecified)	8
Disorders of thyroid gland (i.e. hypothyroidism/hyperthyroidism)	6
Disorders of other endocrine glands (i.e. hypoparathyroidism/hyperparathyroidism)	1
Episodic and paroxysmal disorders (i.e. transient ischaemic attack)	1
Other disorders of the nervous system (unspecified) 1	1
Dementia in other diseases classified elsewhere (i.e. dementia in Parkinson disease)	1
Diseases of liver (unspecified)	1
Disorders of gallbladder, biliary tract and pancreas (i.e. cholelithiasis)	5
Renal failure (unspecified)	1
Soft tissue disorders (i.e. rheumatism, unspecified)	2
Tuberculosis	1
Malignant neoplasms (excl. basal cell carcinoma)	7

For peer review only

For peer review only - http://bmjopen.bmj.com/site/about/guidelines.xhtml

Comorbidities with a prevalence of ≥5% were included in steps 2-4 of the I3-S strategy. In addition to comorbidities, the potential adverse effects of ovarian cancer and its treatment were added to the inventory. These adverse effects were derived from the literature[46], guidelines[47,48] and expert meetings with (gynaecologic) oncologists. We included the adverse effects (incidence of ≥1%) of carboplatin and paclitaxel as these are currently the standard chemotherapy treatments of ovarian cancer.[4] In addition, we included all potential adverse effects of surgery and ovarian cancer itself. We focused on potential adverse effects relevant for health-care providers delivering the exercise or dietary interventions. As a consequences, we excluded descriptions of acute adverse effects of chemotherapy monitored by the treating physicians during admission to the hospital. Additionally, we checked overlap of comorbidities and adverse effects of ovarian cancer and its treatment with previously published I3-S papers for knee osteoarthritis[44] and breast cancer[43] or nutritional guidelines.[37-39,41] The following comorbidities (i.e. hypertensive diseases, ischaemic heart diseases, other forms of heart disease, chronic lower respiratory diseases, Diabetes Mellitus), and adverse effects (clinical parameters such as leukopenia/neutropenia, trombopenia, anemia; and symptoms of dyspnea, nausea, vomiting or diarrhea, skin and nail changes, fever, dizziness, decreased or increased heart rate, change in body weight, depression, numbness/loss of sensation, hearing and/or visual impairments, fatigue, pain and chest pain) were described in previous publications[43,44] or nutritional guidelines[37-39,41] and will not be included in this paper.

In the second and third step, contraindications and restrictions on exercise training were gathered as well as solutions for the exercise training in ovarian cancer specific comorbidities, adverse effects of ovarian cancer and its treatment. This was based on literature[42-44,49], guidelines[38] and/or expert opinions of (gynaecologic) oncologists, physical therapists specialized in oncology.

In the final step, comorbidities, adverse effects of ovarian cancer and its treatment, but also contraindications and restrictions were translated to clinical parameters and symptoms that can be monitored during the intervention of the PADOVA study. All information was synthesized in a framework (Table 2).

For peer review only

207 *Table 2: framework with alternations for the exercise and dietary intervention*

Comorbidities, adverse effects ovarian cancer or treatment	Considerations	Actions/strategy
Comorbidities		
Disorders of thyroid gland (i.e. hypothyroidism/hyperthyroidism)	Consider the following complications of disorders of thyroid gland:<br>• Weight loss or weight gain<br>• Bradycardia in hypothyroidism or tachycardia in hyperthyroidism<br>• Low energy/fatigue in hypothyroidism	• Refer to dietitian when weight loss/-gain occurs<br>• Explain to patient brady-/tachycardia due to hypo-/hyperthyroidism<br>• Monitor symptoms: in case of persistent co-existing symptoms (dyspnea, anxiety, fatigue): terminate exercise and refer to physician<br>• Explain to patient fatigue due to hypothyroidism<br>• Refer to physician when fatigue does not reduce in a few weeks
Disorders of gallbladder, biliary tract and pancreas (i.e. cholelithiasis)		• Monitor symptoms and in case of persistent pain: terminate exercise and refer to physician
Malignant neoplasms (excl. basal cell carcinoma) [1]	Consider comorbidities and adverse effects of the malignant neoplasm that might interfere with the intervention	
Adverse effects ovarian cancer		
Ascites (60%)	Consider the following complications of ascites:<br>• Discomfort<br>• Pleural effusion and/or shortness in breath<br>• Diminished nutritional intake	• Adjust exercise to a comfortable intensity or posture<br>• In case of disproportional dyspnea: terminate exercise session and refer to physician<br>• Refer to dietitian
Adverse effects of chemotherapy and surgery		
Abdominal wound	Consider the following complications of an abdominal wound:	• In the post-operative period (4-6 weeks) exercise is allowed, but pressure on the abdomen should be avoided therefore the 'abdominal crunch' and 'pullover' could be replaced with a 'lateral raise' and 'leg extension'<br>• After 4-6 weeks isometric exercises could be replaced by eccentric exercises<br>• Monitor symptoms: in case of pain or discomfort decrease training intensity or resistance

For peer review only - http://bmjopen.bmj.com/site/about/guidelines.xhtml

	• Fever >38.5 degrees Celsius due to wound infection	• Contra-indication for exercise, refer to physician
Intestinal stoma	Consider the following complications of an intestinal stoma<br>• High output stoma (production of >1 liter per 24 hour during ≥ 3 days)	• See recommendation under 'abdominal wound'<br>• Avoid contact sport (e.g. football or martial arts)<br>• Contra-indication for exercise, refer to physician and/or dietitian
(Risk of) lymphedema in legs	Consider the following complications of lymphedema in legs:<br>• Numbness/loss of sensation	• Monitor leg volume during exercise program; ask for symptoms of lymphedema<br>• Refer to lymphedema specialist when lymphedema is present, or when leg volumes increases and symptoms arise (advice on how to progress with exercise)<br>• Be careful with exercises that include walking, running or balance to prevent falls<br>• Advise patient to wear good fitting, stable footwear with good grip under surface
Deep vein thrombosis (in leg)		• Contra-indication for exercise, refer to a physician when the following symptoms occur: pain in the leg, red or discolored skin, or a feeling of warmth
Thrombophlebitis		• Avoid pressure or impact on affected area<br>• Monitor symptoms and in case of pain or discomfort decrease training intensity or resistance
Nervousness/confusion	Consider the following cause of nervousness/confusion<br>• Sever anxiety or a psychiatric disorder	• Give the patient time to discuss feelings or thoughts<br>• Contra-indication for exercise, refer to a physician when a serious psychiatric disorder might be present
Gastro-intestinal symptoms (i.e. anorexia, dyspepsia, constipation, taste disorder, dry mouth, mouth ulcers, stomatitis)	Consider the following complications of gastro-intestinal symptoms:<br>• Diminished nutritional intake	• Refer to dietitian and/or physician
Melaena		• Contra-indication for exercise, refer to a physician

[1] *Patients with a current other malignancy or prior diagnosis (within 5 years) of cancer were excluded from participation in the PADOVA study*

For peer review only - http://bmjopen.bmj.com/site/about/guidelines.xhtml

Exercise intervention

The exercise intervention consists of two one-hour exercise sessions per week including moderate-to-high intensity resistance and aerobic exercises. The exercise sessions are supervised by a physical therapist specifically trained in treating oncology patients.

The sessions start with a warming up of 10 minutes. Resistance exercises targeting six large muscle groups are conducted for 20 minutes per session, with two series of eight repetitions at 70-80% (gradually increasing per week in between one repetition maximum testing) of the one repetition maximum. Prescribed exercises include vertical row, leg press, bench press, pull over, abdominal crunch and lunge. However, due to the abdominal wound in the post-operative period (4-6 weeks) patients are not allowed to perform eccentric exercises with the abdominal muscles. Additionally, patients are not allowed to put pressure on the abdomen and should therefore avoid heavy lifting and exercises such as the abdominal crunch and pullover. Exercises with an isometric use of the abdominal muscles such as a lateral raise or leg extensions, are an alternative. An overview of all adaptations for patients with ovarian cancer are shown is Table 2. One repetition maximum testing will be repeated every three weeks to ensure adequate training intensity. Aerobic exercises are conducted for 30 minutes per session, with an intensity of 50-80% of the maximal work load as estimated by the steep ramp test[50] and if needed adjusted if the Borg Scale of perceived exertion decreases to a score of 12 or lower or increases to a score of 16 or higher.[51] In addition to the supervised sessions, patients are encouraged to be physically active on at least three additional days a week for 30 minutes to meet the recommended physical activity levels.[40]

Dietary intervention

The dietary intervention is based on the dietary guideline set by the World Cancer Research Fund (WCRF)/American Institute for Cancer Research (AICR)[40] and an sufficient total protein intake of at least 1.2 grams of protein per kg body weight per day[41,52] and 25 grams of protein per meal, since such evenly distributed protein intake is expected to optimize muscle protein synthesis.[53] The intervention is provided by experienced oncology dietitians once every three weeks during face-to-face sessions of 30-45 minutes each at the hospital or by telephone using motivational interviewing techniques.[54] During the first session, patients will receive feedback on their body weight, Body Mass Index (BMI), body composition assessed via bio-electric impedance analysis (BIA), diet quality, and on the extent to which they meet the protein goals and WCRF/AICR dietary recommendations. Counselling

For peer review only - http://bmjopen.bmj.com/site/about/guidelines.xhtml

is tailored to the nutritional needs of each individual patient according to body composition, nutritional status and dietary intake during chemotherapy. Patients who are (at risk of) malnutrition are primarily counselled for prevention of weight loss by maintaining sufficient caloric intake, particularly protein intake. Patients who are not at risk of developing malnutrition are primarily counselled to meet the dietary guidelines set by the WCRF/AICR.

Control group

Women in the control group will receive usual care during chemotherapy, which does not include structured exercise and/or dietary counselling. They are offered a maximum of three exercise and three dietary counselling sessions in twelve weeks after completion of chemotherapy.

Outcome measurements

An investigator blinded from group allocation conducts measurements at three time points. Participants are instructed not to reveal their group allocation. Baseline measurements are conducted before randomisation and the start of chemotherapy (T0), the second measurement three weeks after completion of chemotherapy (T1) and the last measurement (T2) twelve weeks later. An overview of all outcome measurements is presented in Table 3.

Table 3: Summary of outcome measurements

Outcome	Instrument	T0	T1	T2
Primary outcomes				
Body composition	Computed tomography imaging for the assessment of skeletal muscle area	X	X	
	Bio-electric impedance analysis for the assessment of fat mass	X	X	X
Physical fatigue	Multidimensional Fatigue Inventory[1]	X	X	X
Physical function	European Organisation Research and Treatment of Cancer-Quality of life questionnaire-Core 30[2]	X	X	X
Secondary outcomes				
Physical activity	Accelerometer (Actigraph) for objective measurement of physical activity in seven consecutive days during all waking hours	X	X	X
	Physical Activity Scale for the Elderly[3] for self-reported levels of physical activity	X	X	X
Physical fitness	Maximum oxygen uptake (peak VO_2) during a maximum exercise test on a cycle ergometer using a ramp protocol for the assessment of cardiorespiratory fitness	X	X	X
	Hand held dynamometer for the assessment of muscle strength	X	X	X

For peer review only - http://bmjopen.bmj.com/site/about/guidelines.xhtml

Body Mass Index	Body height on a calibrated scale to the nearest 0.1 mm	X	X	X
	Body weight on a calibrated scale to the nearest 0.1 kg	X	X	X
Dietary intake; WCRF/AICR guidelines	Food frequency questionnaire (developed by Wageningen University)	X	X	X
Dietary intake; protein intake	Self-composed food frequency questionnaire	X	X	X
Health-related quality of life and symptoms	European Organisation Research and Treatment of Cancer-Quality of life questionnaire – Ovarian cancer (OV28)[4]	X	X	X
	European Organisation Research and Treatment of Cancer-Quality of life questionnaire - Chemotherapy-induced peripheral neuropathy (CIPN20)[5]	X	X	X
	Hospital Anxiety and Depression Scale[6]	X	X	X
	Pittsburgh Sleep Quality Index[7]	X	X	X
Chemotherapy therapy completion rates and treatment toxicity	Medical records of which chemotherapy completion rates will be assesses as the relative dose intensity, i.e., the amount of particular chemotherapy given in relation to the originally planned chemotherapy dose.		X	
Other study parameters				
Blood sampling	Venous blood sample (40 ml)	X	X	
Smoking and sociodemographics	Self-composed questionnaire	X		
Contamination of control group[1]	Self-composed questionnaire		X	

[1] *Parameter will only be assessed in the control group*

Primary outcomes

Primary outcomes are body composition, physical function and fatigue. Body composition is assessed via skeletal muscle area and fat mass. Skeletal muscle area is assessed at T0 and T1 using routine computed tomography (CT) imaging (first image extending from the third lumbar vertebra to iliac crest) conducted for diagnostic purposes. CT is considered the gold standard for assessment of muscle mass in cancer patients.[55] Because the CT of the third lumbar vertebra is not yet validated for the assessment of body fat mass,[56] fat mass is assessed with a non-invasive measurement BIA.[57] Physical function is assessed using the physical function subscale of the validated European Organisation Research and Treatment of Cancer-Quality of life questionnaire-Core 30 (EORTC QLQ-C30).[58] Physical fatigue is assessed with the physical fatigue subscale of the Multidimensional Fatigue Inventory (MFI).[59]

Secondary outcomes

For peer review only - http://bmjopen.bmj.com/site/about/guidelines.xhtml

Secondary outcomes are physical activity, physical fitness (i.e. cardiorespiratory fitness and muscle strength), dietary intake, BMI, health-related quality of life and symptoms of neuropathy, anxiety and depression, sleep disturbances, chemotherapy treatment toxicity and completion rates. The measurement instruments for the assessment of the secondary outcomes are presented in Table 3.

Other study parameters

In addition, during the visit on T0 and T1, a venous blood sample is drawn and stored in a biobank for future biomarker studies. Covariates such as clinical data (e.g. cancer subtype, FIGO stage) and sociodemographic characteristics (e.g. age, ethnicity) are assessed at baseline. Contamination (i.e. received supervised exercise and/or dietary counselling) of the control group is assessed by a questionnaire at T1.

Process evaluation

An extensive process evaluation is conducted using a mixed-method approach (using both quantitative and qualitative research methods). Quantitative data on behavioral counselling skills of dietitians will be assessed during counselling sessions with the Behavior Change Counselling Inventory (BECCI).[60] In patients in the intervention group, the Health Care Climate Questionnaire (HCCQ) will additionally be used to assess the extent to which an autonomy supportive environment was perceived during counseling.[61] Quantitative data on potential mediating effects of behavioral determinants of physical activity and dietary behavior (i.e. outcome expectations, self-efficacy for eating and exercise habits, sociostructural factors, stage of change, type of motivation and knowledge) are assessed on T0, T1 and T2 by questionnaires in all patients. The following process evaluation components are examined by physical therapists and dietitians and involved in the exercise or dietary intervention via a report form: dose delivered, dose received and fidelity. Acceptability of the intervention is assessed by a questionnaire in patients and a semi-structured interview in patients, physical therapists and dietitians. The semi-structured interviews in patients and healthcare professionals are conducted by a researcher. Interviews will be transcribed verbatim, coded in several phases[62,63] and then analyzed with a qualitative data analysis program. Analyses are partly performed concurrently with data collection because the interviews will be held until data saturation is reached. An overview of all measurement instruments for the process evaluation is presented in Table 4.

For peer review only - http://bmjopen.bmj.com/site/about/guidelines.xhtml

Table 4: Overview of all measurement instruments for the process evaluation

Outcome	Instrument	T0	T1	T2
Process evaluation				
Behavioral determinants	Multidimensional Outcome Expectations for Exercise Scale[3]	X	X	X
	Self-efficacy for eating and exercise habits – self-composed items	X	X	X
	Sociostructural factors, self-composed items	X	X	X
	Stage of change[8]	X	X	X
	Type of motivation: Treatment Self-Regulation Questionnaire[9]	X	X	X
	Knowledge (on WCRF recommendations): Items used in previous study[10]	X	X	X
Acceptability of intervention	Self-composed items[1]		X	
	The Health Care Climate Questionnaire – short form (6 items)[11]		X	
Acceptability of intervention	Semi-structured interviews in healthcare professionals[2]		X[1]	X[4]
	Semi-structured interviews in participants		X[1]	X[4]
Dose delivered	Checklist[2] – Amount of (components of) sessions provided by physical therapist/dietitian	Daily, during intervention		
Dose received	Checklist[2] – Amount of (components of) sessions participants actively engaged in	Daily, during intervention		
Fidelity	Checklist[2] – Extend to which the intervention was executed as prescribed in the protocol	Daily, during intervention		
Behavioral counselling skills[3]	Behavior Change Counselling Inventory[12]	During intervention		

[1] Parameters will only be assessed in intervention group
[2] Parameter will be examined in physical therapists and dietitians
[3] Parameter will be examined in dietitians
[4] Parameter will only be assessed in the control group

For peer review only

Sample size calculation

Sample size calculation was based on the results of a previous RCT among patients with breast cancer that evaluated the effects of a combined aerobic and resistance exercise intervention (similar to the PADOVA study),[28] and a pilot study among patients with ovarian cancer that evaluated the feasibility of an exercise intervention.[34] With 53 patients per study-arm, we are able to detect a clinically relevant between group difference in effects directly post-intervention on physical function (10 points), physical fatigue (2.7 points), body composition (effect size 0.55 in percentage body fat), and 10-15% difference in peak oxygen uptake[64] (alpha=0.05; power= 0.80). Taking into account a dropout of 15%, we aim to include 61 patients per group.

Statistical analysis

The primary analysis focuses on the effects of the intervention directly after completion of chemotherapy (T1), since both the intervention and control group have received counselling from a physical therapist

For peer review only - http://bmjopen.bmj.com/site/about/guidelines.xhtml

and/or dietitian at follow-up (T2). Data will be analysed according to the intention-to-treat principle. Intervention effects (at T1) will be assessed using linear regression analysis for continuous outcomes, by regressing the intervention on post-test value (T1) of the outcome, adjusting for baseline values (T0). To examine whether intervention effects on body composition, physical function or fatigue are mediated by changes in physical activity and fitness, and/or diet, a series of regression analysis according to the product-of-coefficients test will be conducted.[65] Potential effect modification by relevant demographic (e.g. age) and clinical (e.g. cancer stage, treatment regimen) variables will be explored by adding the variable and its interaction term with the intervention into the regression model.

DISCUSSION

This paper presents the rationale and design of the PADOVA study and the procedure of tailoring an exercise and dietary intervention to patients with ovarian cancer. The PADOVA study aims to examine the effectiveness of a combined exercise and dietary intervention on body composition, physical function and fatigue in patients with ovarian cancer during chemotherapy. Additionally, during the PADOVA study, an extensive process evaluation is conducted to examine the effective and ineffective components of the intervention. This large randomised controlled trial significantly contributes to the scientific evidence on the potential benefits of exercise and dietary support in an understudied group of patients with cancer who often face a complex and unfavourable disease trajectory.

A strength of this study is its randomized controlled design in a relatively large group of women with ovarian cancer and the systematic development of the exercise and dietary intervention by the I3-S strategy to ensure adequate tailoring of the intervention to this patient specific group. Because the intervention is adjusted to comorbidities, disease- and treatment induced effects, we expect compliance to the intervention to be high and drop out to be low. Another strength is the process evaluation because it retrieves information on how and why the intervention is (un)successful.[66] With this information the exercise and dietary intervention will be improved before the combined intervention is implemented in clinical practice.

Aiming to maximize benefits on body composition, we combined an exercise and dietary intervention. Consequently, we are unable to disentangle the effects of the exercise and dietary intervention. However, we plan to perform a mediation analysis to explore whether the intervention effects on body composition can be explained by changes in exercise or dietary components. Another limitation of this study is its inability to study long-term effects of the intervention compared to the control group. In the PADOVA study, exercise and dietary counselling will be offered to the control group after the first follow up in order to prevent nonparticipation and dropout and to limit contamination (exercise and dietary intervention) in the control group during chemotherapy. Therefore we expect smaller differences between groups at follow-up (T2).

This study will contribute to the evidence on the benefits of an exercise and dietary intervention in patients with ovarian cancer. If proven effective, a combined exercise and dietary intervention for patients with ovarian cancer can be implemented in clinical practice.

Patient and public involvement

For peer review only - http://bmjopen.bmj.com/site/about/guidelines.xhtml

Patients were involved in the development of study specific patient information and they were asked to assess the burden of time required to participate in this study. The opinion of patients has been considered to improve the readability of the patient information sheets on the study. During the study patients will be interviewed, as part of the process evaluation, to improve the intervention. This information could be important for implementation of the intervention in clinical practice. Study results will be presented to patients in collaboration with the patient community.

Ethics and dissemination

This study has been approved by the medical ethical committee of the Amsterdam UMC (reference: 018). Additional approval was obtained for the participating hospitals. The trial is registered in the Netherlands Trial Register. Signed informed consent is required of all included participants. Results of the study will be published in international peer-reviewed journals.

Acknowledgements

Not applicable.

Contributors

EK, GGK, LMB and MH conceived the study. GGK, LMB, LRCWL, MH and SS designed the study. LMB, MH, RJH and SS designed the intervention. LMB, MH and SS wrote the manuscript. All authors read and approved the final manuscript.

Funding

The PADOVA study is funded by the Dutch Cancer Society, grant number VU 2015-7950. The Dutch Cancer Society was not involved in the design of the study, the collection, analysis and interpretation of data, nor in writing the manuscript.

Competing interests

The authors declare that they have no competing interests.

Patient consent for publication

Not required.

For peer review only - http://bmjopen.bmj.com/site/about/guidelines.xhtml

Provenance and peer review

Not commissioned; externally peer reviewed.

REFERENCES

1. Ferlay J, Soerjomataram I, Dikshit R, et al. Cancer incidence and mortality worldwide: sources, methods and major patterns in GLOBOCAN 2012. *Int J Cancer* 2015;136(5):E359-86. doi: 10.1002/ijc.29210 [published Online First: 2014/09/16]
2. Reid BM, Permuth JB, Sellers TA. Epidemiology of ovarian cancer: a review. *Cancer Biol Med* 2017;14(1):9-32.
3. Netherlands CCCt. Epithelial Ovarian Carcinoma; National guidelines 2.0. 2012
4. Group obotEGW, Raja FA, Ledermann JA, et al. Newly diagnosed and relapsed epithelial ovarian carcinoma: ESMO Clinical Practice Guidelines for diagnosis, treatment and follow-up†. *Annals of Oncology* 2013;24(suppl_6):vi24-vi32. doi: 10.1093/annonc/mdt333
5. Holzner B, Kemmler G, Meraner V, et al. Fatigue in ovarian carcinoma patients: a neglected issue? *Cancer* 2003;97(6):1564-72. doi: 10.1002/cncr.11253 [published Online First: 2003/03/11]
6. Meraner V, Gamper EM, Grahmann A, et al. Monitoring physical and psychosocial symptom trajectories in ovarian cancer patients receiving chemotherapy. *BMC Cancer* 2012;12:77. doi: 10.1186/1471-2407-12-77 [published Online First: 2012/03/01]
7. von Gruenigen VE, Huang HQ, Gil KM, et al. Assessment of factors that contribute to decreased quality of life in Gynecologic Oncology Group ovarian cancer trials. *Cancer* 2009;115(20):4857-64. doi: 10.1002/cncr.24520 [published Online First: 2009/08/20]
8. Kayl AE, Meyers CA. Side-effects of chemotherapy and quality of life in ovarian and breast cancer patients. *Curr Opin Obstet Gynecol* 2006;18(1):24-8. doi: 10.1097/01.gco.0000192996.20040.24 [published Online First: 2006/02/24]
9. Rutten IJ, van Dijk DP, Kruitwagen RF, et al. Loss of skeletal muscle during neoadjuvant chemotherapy is related to decreased survival in ovarian cancer patients. *J Cachexia Sarcopenia Muscle* 2016;7(4):458-66. doi: 10.1002/jcsm.12107 [published Online First: 2016/04/01]
10. Kumar A, Moynagh MR, Multinu F, et al. Muscle composition measured by CT scan is a measurable predictor of overall survival in advanced ovarian cancer. *Gynecol Oncol* 2016;142(2):311-6. doi: 10.1016/j.ygyno.2016.05.027
11. Mardas M, Stelmach-Mardas M, Madry R. Body weight changes in patients undergoing chemotherapy for ovarian cancer influence progression-free and overall survival. *Support Care Cancer* 2017;25(3):795-800. doi: 10.1007/s00520-016-3462-1
12. von Gruenigen VE, Frasure HE, Jenison EL, et al. Longitudinal assessment of quality of life and lifestyle in newly diagnosed ovarian cancer patients: the roles of surgery and chemotherapy. *Gynecol Oncol* 2006;103(1):120-6. doi: 10.1016/j.ygyno.2006.01.059 [published Online First: 2006/03/25]
13. Gil KM, Gibbons HE, Jenison EL, et al. Baseline characteristics influencing quality of life in women undergoing gynecologic oncology surgery. *Health Qual Life Outcomes* 2007;5:25. doi: 10.1186/1477-7525-5-25 [published Online First: 2007/05/19]
14. Wenzel L, Huang HQ, Monk BJ, et al. Quality-of-life comparisons in a randomized trial of interval secondary cytoreduction in advanced ovarian carcinoma: a Gynecologic Oncology Group study. *J Clin Oncol* 2005;23(24):5605-12. doi: 10.1200/jco.2005.08.147 [published Online First: 2005/08/20]
15. Kumar A, Bakkum-Gamez JN, Weaver AL, et al. Impact of obesity on surgical and oncologic outcomes in ovarian cancer. *Gynecol Oncol* 2014;135(1):19-24. doi: 10.1016/j.ygyno.2014.07.103 [published Online First: 2014/08/12]
16. Pavelka JC, Brown RS, Karlan BY, et al. Effect of obesity on survival in epithelial ovarian cancer. *Cancer* 2006;107(7):1520-4. doi: 10.1002/cncr.22194 [published Online First: 2006/08/31]
17. Aust S, Knogler T, Pils D, et al. Skeletal Muscle Depletion and Markers for Cancer Cachexia Are Strong Prognostic Factors in Epithelial Ovarian Cancer. *PLoS One* 2015;10(10):e0140403. doi: 10.1371/journal.pone.0140403
18. Bandera EV, Lee VS, Rodriguez-Rodriguez L, et al. Impact of Chemotherapy Dosing on Ovarian Cancer Survival According to Body Mass Index. *JAMA Oncol* 2015;1(6):737-45. doi: 10.1001/jamaoncol.2015.1796 [published Online First: 2015/07/17]

For peer review only - http://bmjopen.bmj.com/site/about/guidelines.xhtml

19. Kim SI, Kim HS, Kim TH, et al. Impact of underweight after treatment on prognosis of advanced-stage ovarian cancer. *J Immunol Res* 2014;2014:349546. doi: 10.1155/2014/349546 [published Online First: 2014/07/23]
20. Mardas M, Jamka M, Madry R, et al. Dietary habits changes and quality of life in patients undergoing chemotherapy for epithelial ovarian cancer. *Support Care Cancer* 2015;23(4):1015-23. doi: 10.1007/s00520-014-2462-2 [published Online First: 2014/10/02]
21. Laky B, Janda M, Bauer J, et al. Malnutrition among gynaecological cancer patients. *Eur J Clin Nutr* 2007;61(5):642-6. doi: 10.1038/sj.ejcn.1602540
22. Hess LM, Barakat R, Tian C, et al. Weight change during chemotherapy as a potential prognostic factor for stage III epithelial ovarian carcinoma: a Gynecologic Oncology Group study. *Gynecol Oncol* 2007;107(2):260-5. doi: 10.1016/j.ygyno.2007.06.010 [published Online First: 2007/08/07]
23. Schmid D, Leitzmann MF. Cardiorespiratory fitness as predictor of cancer mortality: a systematic review and meta-analysis. *Annals of oncology : official journal of the European Society for Medical Oncology* 2015;26(2):272-8. doi: 10.1093/annonc/mdu250 [published Online First: 2014/07/11]
24. McTiernan A, Friedenreich CM, Katzmarzyk PT, et al. Physical Activity in Cancer Prevention and Survival: A Systematic Review. *Med Sci Sports Exerc* 2019;51(6):1252-61. doi: 10.1249/mss.0000000000001937 [published Online First: 2019/05/17]
25. Padilha CS, Marinello PC, Galvao DA, et al. Evaluation of resistance training to improve muscular strength and body composition in cancer patients undergoing neoadjuvant and adjuvant therapy: a meta-analysis. *J Cancer Surviv* 2017;11(3):339-49. doi: 10.1007/s11764-016-0592-x [published Online First: 2017/01/06]
26. Cohen CW, Fontaine KR, Arend RC, et al. A Ketogenic Diet Reduces Central Obesity and Serum Insulin in Women with Ovarian or Endometrial Cancer. *J Nutr* 2018;148(8):1253-60. doi: 10.1093/jn/nxy119 [published Online First: 2018/08/24]
27. Kampshoff CS, Chinapaw MJ, Brug J, et al. Randomized controlled trial of the effects of high intensity and low-to-moderate intensity exercise on physical fitness and fatigue in cancer survivors: results of the Resistance and Endurance exercise After ChemoTherapy (REACT) study. *BMC Med* 2015;13:275. doi: 10.1186/s12916-015-0513-2 [published Online First: 2015/10/31]
28. van Waart H, Stuiver MM, van Harten WH, et al. Effect of Low-Intensity Physical Activity and Moderate- to High-Intensity Physical Exercise During Adjuvant Chemotherapy on Physical Fitness, Fatigue, and Chemotherapy Completion Rates: Results of the PACES Randomized Clinical Trial. *J Clin Oncol* 2015;33(17):1918-27. doi: 10.1200/jco.2014.59.1081 [published Online First: 2015/04/29]
29. Demark-Wahnefried W, Morey MC, Sloane R, et al. Reach out to enhance wellness home-based diet-exercise intervention promotes reproducible and sustainable long-term improvements in health behaviors, body weight, and physical functioning in older, overweight/obese cancer survivors. *J Clin Oncol* 2012;30(19):2354-61. doi: 10.1200/jco.2011.40.0895 [published Online First: 2012/05/23]
30. Johns DJ, Hartmann-Boyce J, Jebb SA, et al. Diet or exercise interventions vs combined behavioral weight management programs: a systematic review and meta-analysis of direct comparisons. *J Acad Nutr Diet* 2014;114(10):1557-68. doi: 10.1016/j.jand.2014.07.005 [published Online First: 2014/09/27]
31. Jager R, Kerksick CM, Campbell BI, et al. International Society of Sports Nutrition Position Stand: protein and exercise. *J Int Soc Sports Nutr* 2017;14:20.
32. Gil KM, von Gruenigen VE. Physical activity and gynecologic cancer survivorship. *Recent Results Cancer Res* 2011;186:305-15. doi: 10.1007/978-3-642-04231-7_13 [published Online First: 2010/11/30]
33. Newton MJ, Hayes SC, Janda M, et al. Safety, feasibility and effects of an individualised walking intervention for women undergoing chemotherapy for ovarian cancer: a pilot study. *BMC Cancer* 2011;11:389. doi: 10.1186/1471-2407-11-389 [published Online First: 2011/09/09]
34. Mizrahi D, Broderick C, Friedlander M, et al. An Exercise Intervention During Chemotherapy for Women With Recurrent Ovarian Cancer: A Feasibility Study. *Int J Gynecol Cancer* 2015;25(6):985-92. doi: 10.1097/igc.0000000000000460 [published Online First: 2015/04/29]
35. von Gruenigen VE, Frasure HE, Kavanagh MB, et al. Feasibility of a lifestyle intervention for ovarian cancer patients receiving adjuvant chemotherapy. *Gynecol Oncol* 2011;122(2):328-33. doi: 10.1016/j.ygyno.2011.04.043 [published Online First: 2011/05/24]
36. Gezond leven na kanker. In: Fonds SWKO, ed.: Wereld Kanker Onderzoeks Fonds, 2017.
37. Oncoline. Algemene voedings- en dieetbehandeling 2017 [updated 01-03-2017. Available from: https://www.oncoline.nl/voedings-en-dieetbehandeling accessed 20-08-2018 2018.
38. Oncoline. Eierstokkanker 2017 [updated 01-03-2017. Available from: https://www.oncoline.nl/voeding-eierstokkanker.

For peer review only - http://bmjopen.bmj.com/site/about/guidelines.xhtml

39. Oncoline. Ondervoeding bij patienten met kanker 2012 [updated 15-01-2012. Available from: https://www.oncoline.nl/ondervoeding-bij-patienten-met-kanker.
40. World Cancer Research Fund / American Institute for Cancer Research. Diet, Nutrition, Physical Activity and Cancer: a Global Perspective. Continuous Update Project Expert report, 2018.
41. Arends J, Bachmann P, Baracos V, et al. ESPEN guidelines on nutrition in cancer patients. *Clin Nutr* 2016 doi: 10.1016/j.clnu.2016.07.015 [published Online First: 2016/09/18]
42. Dekker J, de Rooij M, van der Leeden M. Exercise and comorbidity: the i3-S strategy for developing comorbidity-related adaptations to exercise therapy. *Disabil Rehabil* 2016;38(9):905-9. doi: 10.3109/09638288.2015.1066451
43. van der Leeden M, Huijsmans RJ, Geleijn E, et al. Tailoring exercise interventions to comorbidities and treatment-induced adverse effects in patients with early stage breast cancer undergoing chemotherapy: a framework to support clinical decisions. *Disabil Rehabil* 2017:1-11. doi: 10.1080/09638288.2016.1260647
44. de Rooij M, van der Leeden M, Avezaat E, et al. Development of comorbidity-adapted exercise protocols for patients with knee osteoarthritis. *Clin Interv Aging* 2014;9:829-42.
45. Organization WH. International statistical classification of diseases and related health problems (10th revision) [updated 2016. 5:[Available from: https://icd.who.int/browse10/2016/en2019.
46. Farmacotherapeutisch Kompas: Zorginstituut Nederland; [Available from: https://farmacotherapeutischkompas.nl accessed 20-09-2017.
47. Oncoline. Epitheliaal ovariumcarcinoom 2.1: Integraal Kankercentrum Nederland; 2018 [updated 2018-01-15.
48. Oncoline. Ascites, versie 2.0: Integraal Kankercentrum Nederland; 2010 [updated 19-01-2010.
49. Klein I, Danzi S. Thyroid Disease and the Heart. *Circulation* 2007;116(15):1725-35. doi: doi:10.1161/CIRCULATIONAHA.106.678326
50. De Backer IC, Schep G, Hoogeveen A, et al. Exercise testing and training in a cancer rehabilitation program: the advantage of the steep ramp test. *Arch Phys Med Rehabil* 2007;88(5):610-6. doi: 10.1016/j.apmr.2007.02.013 [published Online First: 2007/05/01]
51. Borg G. Borg's perceived exertion and pain scales. Champaign, IL, US: Human Kinetics 1998.
52. Nitenberg G, Raynard B. Nutritional support of the cancer patient: issues and dilemmas. *Crit Rev Oncol Hematol* 2000;34(3):137-68. [published Online First: 2000/06/06]
53. Paddon-Jones D, Rasmussen BB. Dietary protein recommendations and the prevention of sarcopenia. *Curr Opin Clin Nutr Metab Care* 2009;12(1):86-90. doi: 10.1097/MCO.0b013e32831cef8b [published Online First: 2008/12/06]
54. Miller WR, Rollnick S. Ten things that motivational interviewing is not. *Behav Cogn Psychother* 2009;37(2):129-40. doi: 10.1017/s1352465809005128 [published Online First: 2009/04/15]
55. Prado CM, Birdsell LA, Baracos VE. The emerging role of computerized tomography in assessing cancer cachexia. *Curr Opin Support Palliat Care* 2009;3(4):269-75. doi: 10.1097/SPC.0b013e328331124a [published Online First: 2009/08/12]
56. Shen W, Punyanitya M, Wang Z, et al. Total body skeletal muscle and adipose tissue volumes: estimation from a single abdominal cross-sectional image. *J Appl Physiol (1985)* 2004;97(6):2333-8. doi: 10.1152/japplphysiol.00744.2004 [published Online First: 2004/08/18]
57. Bolanowski M, Nilsson BE. Assessment of human body composition using dual-energy x-ray absorptiometry and bioelectrical impedance analysis. *Medical science monitor : international medical journal of experimental and clinical research* 2001;7(5):1029-33. [published Online First: 2001/09/06]
58. Aaronson NK, Ahmedzai S, Bergman B, et al. The European Organization for Research and Treatment of Cancer QLQ-C30: a quality-of-life instrument for use in international clinical trials in oncology. *J Natl Cancer Inst* 1993;85(5):365-76. [published Online First: 1993/03/03]
59. Smets EM, Garssen B, Bonke B, et al. The Multidimensional Fatigue Inventory (MFI) psychometric qualities of an instrument to assess fatigue. *J Psychosom Res* 1995;39(3):315-25. [published Online First: 1995/04/01]
60. Lane C, Huws-Thomas M, Hood K, et al. Measuring adaptations of motivational interviewing: the development and validation of the behavior change counseling index (BECCI). *Patient Educ Couns* 2005;56(2):166-73. doi: 10.1016/j.pec.2004.01.003 [published Online First: 2005/01/18]
61. Czajkowska Z, Wang H, Hall NC, et al. Validation of the English and French versions of the Brief Health Care Climate Questionnaire. *Health Psychol Open* 2017;4(2):2055102917730675.
62. Boeije H. Analysis in qualitative research: Sage 2009.
63. Green J, Thorogood N. Qualitative methods for health research: Sage 2013.
64. Jones LW, Liang Y, Pituskin EN, et al. Effect of exercise training on peak oxygen consumption in patients with cancer: a meta-analysis. *Oncologist* 2011;16(1):112-20. doi: 10.1634/theoncologist.2010-0197 [published Online First: 2011/01/08]

For peer review only - http://bmjopen.bmj.com/site/about/guidelines.xhtml

65. MacKinnon DP, Fairchild AJ, Fritz MS. Mediation analysis. *Annual review of psychology* 2007;58:593-614. doi: 10.1146/annurev.psych.58.110405.085542 [published Online First: 2006/09/14]
66. Process evaluation for public health interventions and research. San Francisco, CA, US: Jossey-Bass 2002.

REFERECENS IN TABLES

1. Smets EM, Garssen B, Bonke B, et al. The Multidimensional Fatigue Inventory (MFI) psychometric qualities of an instrument to assess fatigue. *J Psychosom Res* 1995;39(3):315-25. [published Online First: 1995/04/01]
2. Aaronson NK, Ahmedzai S, Bergman B, et al. The European Organization for Research and Treatment of Cancer QLQ-C30: a quality-of-life instrument for use in international clinical trials in oncology. *J Natl Cancer Inst* 1993;85(5):365-76. [published Online First: 1993/03/03]
3. Wojcicki TR, White SM, McAuley E. Assessing outcome expectations in older adults: the multidimensional outcome expectations for exercise scale. *J Gerontol B Psychol Sci Soc Sci* 2009;64(1):33-40. doi: 10.1093/geronb/gbn032 [published Online First: 2009/02/03]
4. Greimel E, Bottomley A, Cull A, et al. An international field study of the reliability and validity of a disease-specific questionnaire module (the QLQ-OV28) in assessing the quality of life of patients with ovarian cancer. *Eur J Cancer* 2003;39(10):1402-8. [published Online First: 2003/06/27]
5. Postma TJ, Aaronson NK, Heimans JJ, et al. The development of an EORTC quality of life questionnaire to assess chemotherapy-induced peripheral neuropathy: the QLQ-CIPN20. *Eur J Cancer* 2005;41(8):1135-9. doi: 10.1016/j.ejca.2005.02.012 [published Online First: 2005/05/25]
6. Zigmond AS SR. The hospital anxiety and depression scale. *Acta Psychiatr Scand* 1983;67(6):361-70.
7. Buysse DJ, Reynolds CF, Monk TH, et al. The Pittsburgh sleep quality index: A new instrument for psychiatric practice and research. *Psychiatry Research* 1989;28(2):193-213. doi: https://doi.org/10.1016/0165-1781(89)90047-4
8. Prochaska JO, Velicer WF. The transtheoretical model of health behavior change. *Am J Health Promot* 1997;12(1):38-48. doi: 10.4278/0890-1171-12.1.38 [published Online First: 1997/08/05]
9. Levesque CS, Williams GC, Elliot D, et al. Validating the theoretical structure of the Treatment Self-Regulation Questionnaire (TSRQ) across three different health behaviors. *Health Educ Res* 2007;22(5):691-702. doi: 10.1093/her/cyl148 [published Online First: 2006/12/02]
10. Vrieling A, Visser A, Hoedjes M, et al. Increasing awareness and knowledge of lifestyle recommendations for cancer prevention in Lynch syndrome carriers: Randomized controlled trial. *Clin Genet* 2018;93(1):67-77. doi: 10.1111/cge.13076 [published Online First: 2017/06/21]
11. Czajkowska Z, Wang H, Hall NC, et al. Validation of the English and French versions of the Brief Health Care Climate Questionnaire. *Health Psychol Open* 2017;4(2):2055102917730675.
12. Lane C, Huws-Thomas M, Hood K, et al. Measuring adaptations of motivational interviewing: the development and validation of the behavior change counseling index (BECCI). *Patient Educ Couns* 2005;56(2):166-73. doi: 10.1016/j.pec.2004.01.003 [published Online First: 2005/01/18]

For peer review only

For peer review only - http://bmjopen.bmj.com/site/about/guidelines.xhtml

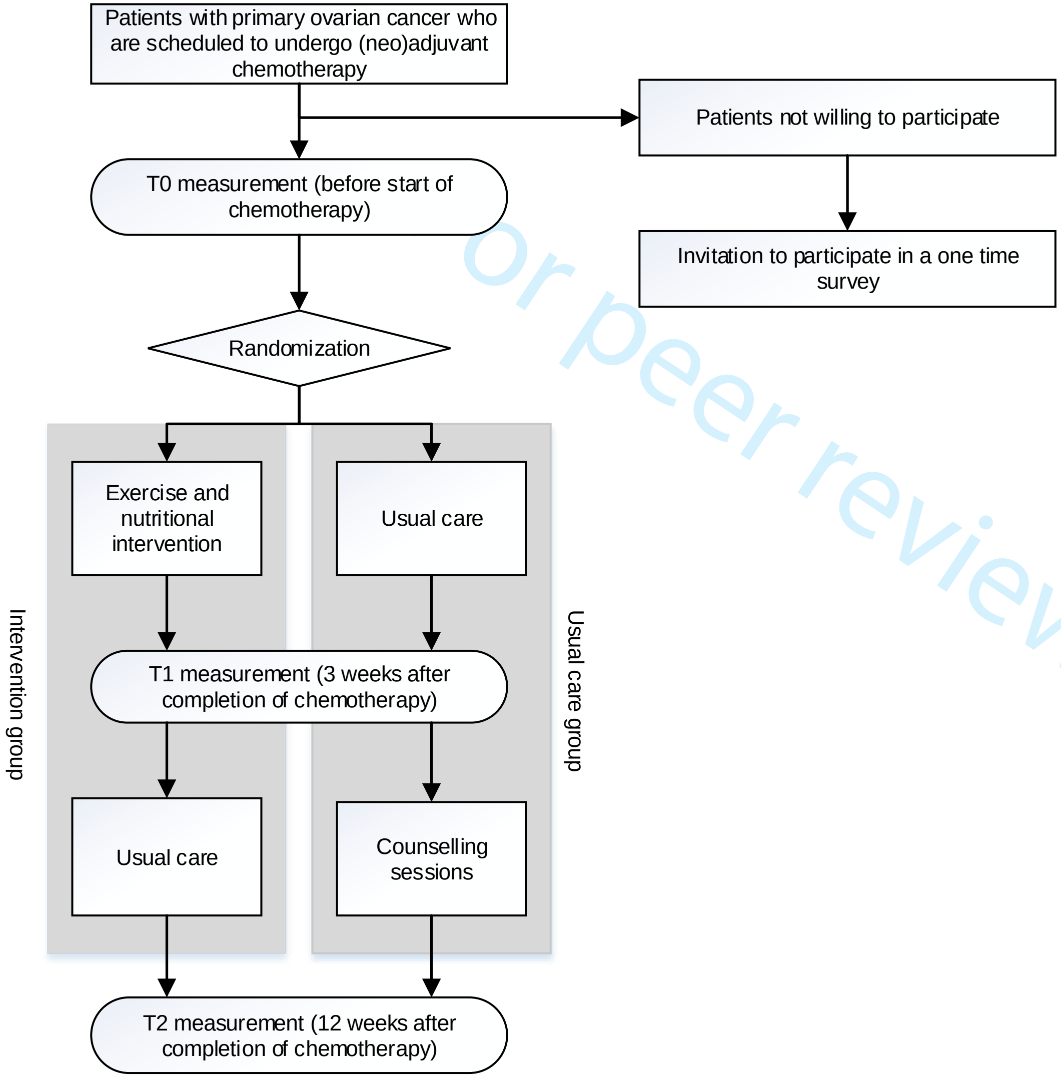

For peer review only - http://bmjopen.bmj.com/site/about/guidelines.xhtml

STANDARD PROTOCOL ITEMS: RECOMMENDATIONS FOR INTERVENTIONAL TRIALS

SPIRIT 2013 Checklist: Recommended items to address in a clinical trial protocol and related documents*

Section/item	Item No	Description	Addressed on page number
**Administrative information**			
Title	1	Descriptive title identifying the study design, population, interventions, and, if applicable, trial acronym	1
Trial registration	2a	Trial identifier and registry name. If not yet registered, name of intended registry	3
	2b	All items from the World Health Organization Trial Registration Data Set	N/A
Protocol version	3	Date and version identifier	In MEC protocol
Funding	4	Sources and types of financial, material, and other support	18
Roles and responsibilities	5a	Names, affiliations, and roles of protocol contributors	1, 18
	5b	Name and contact information for the trial sponsor	1
	5c	Role of study sponsor and funders, if any, in study design; collection, management, analysis, and interpretation of data; writing of the report; and the decision to submit the report for publication, including whether they will have ultimate authority over any of these activities	18
	5d	Composition, roles, and responsibilities of the coordinating centre, steering committee, endpoint adjudication committee, data management team, and other individuals or groups overseeing the trial, if applicable (see Item 21a for data monitoring committee)	N/A

For peer review only

**Introduction**			
Background and rationale	6a	Description of research question and justification for undertaking the trial, including summary of relevant studies (published and unpublished) examining benefits and harms for each intervention	4,5
	6b	Explanation for choice of comparators	4,5
Objectives	7	Specific objectives or hypotheses	4,5
Trial design	8	Description of trial design including type of trial (eg, parallel group, crossover, factorial, single group), allocation ratio, and framework (eg, superiority, equivalence, noninferiority, exploratory)	5,6
**Methods: Participants, interventions, and outcomes**			
Study setting	9	Description of study settings (eg, community clinic, academic hospital) and list of countries where data will be collected. Reference to where list of study sites can be obtained	6
Eligibility criteria	10	Inclusion and exclusion criteria for participants. If applicable, eligibility criteria for study centres and individuals who will perform the interventions (eg, surgeons, psychotherapists)	6
Interventions	11a	Interventions for each group with sufficient detail to allow replication, including how and when they will be administered	6-13
	11b	Criteria for discontinuing or modifying allocated interventions for a given trial participant (eg, drug dose change in response to harms, participant request, or improving/worsening disease)	6-13
	11c	Strategies to improve adherence to intervention protocols, and any procedures for monitoring adherence (eg, drug tablet return, laboratory tests)	6-15
	11d	Relevant concomitant care and interventions that are permitted or prohibited during the trial	N/A
Outcomes	12	Primary, secondary, and other outcomes, including the specific measurement variable (eg, systolic blood pressure), analysis metric (eg, change from baseline, final value, time to event), method of aggregation (eg, median, proportion), and time point for each outcome. Explanation of the clinical relevance of chosen efficacy and harm outcomes is strongly recommended	13-16
Participant timeline	13	Time schedule of enrolment, interventions (including any run-ins and washouts), assessments, and visits for participants. A schematic diagram is highly recommended (see Figure)	6-15, figure 1, table 3 and 4

For peer review only - http://bmjopen.bmj.com/site/about/guidelines.xhtml

Sample size	14	Estimated number of participants needed to achieve study objectives and how it was determined, including clinical and statistical assumptions supporting any sample size calculations	16
Recruitment	15	Strategies for achieving adequate participant enrolment to reach target sample size	In MEC protocol
**Methods: Assignment of interventions (for controlled trials)**			
Allocation:			
Sequence generation	16a	Method of generating the allocation sequence (eg, computer-generated random numbers), and list of any factors for stratification. To reduce predictability of a random sequence, details of any planned restriction (eg, blocking) should be provided in a separate document that is unavailable to those who enrol participants or assign interventions	6
Allocation concealment mechanism	16b	Mechanism of implementing the allocation sequence (eg, central telephone; sequentially numbered, opaque, sealed envelopes), describing any steps to conceal the sequence until interventions are assigned	6
Implementation	16c	Who will generate the allocation sequence, who will enrol participants, and who will assign participants to interventions	6
Blinding (masking)	17a	Who will be blinded after assignment to interventions (eg, trial participants, care providers, outcome assessors, data analysts), and how	13
	17b	If blinded, circumstances under which unblinding is permissible, and procedure for revealing a participant's allocated intervention during the trial	N/A
**Methods: Data collection, management, and analysis**			
Data collection methods	18a	Plans for assessment and collection of outcome, baseline, and other trial data, including any related processes to promote data quality (eg, duplicate measurements, training of assessors) and a description of study instruments (eg, questionnaires, laboratory tests) along with their reliability and validity, if known. Reference to where data collection forms can be found, if not in the protocol	13-16
	18b	Plans to promote participant retention and complete follow-up, including list of any outcome data to be collected for participants who discontinue or deviate from intervention protocols	13-16

Data management	19	Plans for data entry, coding, security, and storage, including any related processes to promote data quality (eg, double data entry; range checks for data values). Reference to where details of data management procedures can be found, if not in the protocol	16-17
Statistical methods	20a	Statistical methods for analysing primary and secondary outcomes. Reference to where other details of the statistical analysis plan can be found, if not in the protocol	16-17
	20b	Methods for any additional analyses (eg, subgroup and adjusted analyses)	16-17
	20c	Definition of analysis population relating to protocol non-adherence (eg, as randomised analysis), and any statistical methods to handle missing data (eg, multiple imputation)	16-17
**Methods: Monitoring**			
Data monitoring	21a	Composition of data monitoring committee (DMC); summary of its role and reporting structure; statement of whether it is independent from the sponsor and competing interests; and reference to where further details about its charter can be found, if not in the protocol. Alternatively, an explanation of why a DMC is not needed	Low risk study, therefore DMC is not needed
	21b	Description of any interim analyses and stopping guidelines, including who will have access to these interim results and make the final decision to terminate the trial	N/A
Harms	22	Plans for collecting, assessing, reporting, and managing solicited and spontaneously reported adverse events and other unintended effects of trial interventions or trial conduct	N/A
Auditing	23	Frequency and procedures for auditing trial conduct, if any, and whether the process will be independent from investigators and the sponsor	N/A
**Ethics and dissemination**			
Research ethics approval	24	Plans for seeking research ethics committee/institutional review board (REC/IRB) approval	5-6
Protocol amendments	25	Plans for communicating important protocol modifications (eg, changes to eligibility criteria, outcomes, analyses) to relevant parties (eg, investigators, REC/IRBs, trial participants, trial registries, journals, regulators)	In MEC protocol

For peer review only - http://bmjopen.bmj.com/site/about/guidelines.xhtml

Consent or assent	26a	Who will obtain informed consent or assent from potential trial participants or authorised surrogates, and how (see Item 32)	In MEC protocol
	26b	Additional consent provisions for collection and use of participant data and biological specimens in ancillary studies, if applicable	N/A
Confidentiality	27	How personal information about potential and enrolled participants will be collected, shared, and maintained in order to protect confidentiality before, during, and after the trial	In MEC protocol
Declaration of interests	28	Financial and other competing interests for principal investigators for the overall trial and each study site	18
Access to data	29	Statement of who will have access to the final trial dataset, and disclosure of contractual agreements that limit such access for investigators	In MEC protocol
Ancillary and post-trial care	30	Provisions, if any, for ancillary and post-trial care, and for compensation to those who suffer harm from trial participation	In MEC protocol
Dissemination policy	31a	Plans for investigators and sponsor to communicate trial results to participants, healthcare professionals, the public, and other relevant groups (eg, via publication, reporting in results databases, or other data sharing arrangements), including any publication restrictions	In MEC protocol
	31b	Authorship eligibility guidelines and any intended use of professional writers	N/A
	31c	Plans, if any, for granting public access to the full protocol, participant-level dataset, and statistical code	N/A
**Appendices**			
Informed consent materials	32	Model consent form and other related documentation given to participants and authorised surrogates	In IRB approval
Biological specimens	33	Plans for collection, laboratory evaluation, and storage of biological specimens for genetic or molecular analysis in the current trial and for future use in ancillary studies, if applicable	N/A

*It is strongly recommended that this checklist be read in conjunction with the SPIRIT 2013 Explanation & Elaboration for important clarification on the items. Amendments to the protocol should be tracked and dated. The SPIRIT checklist is copyrighted by the SPIRIT Group under the Creative Commons "Attribution-NonCommercial-NoDerivs 3.0 Unported" license.

For peer review only - http://bmjopen.bmj.com/site/about/guidelines.xhtml

BMJ Open

Rationale and study protocol of the Physical Activity and Dietary intervention in women with OVArian cancer (PADOVA) study: A randomised controlled trial to evaluate effectiveness of a tailored exercise and dietary intervention on body composition, physical function and fatigue in women with ovarian cancer undergoing chemotherapy

Journal:	*BMJ Open*
Manuscript ID	bmjopen-2020-036854.R1
Article Type:	Protocol
Date Submitted by the Author:	27-May-2020
Complete List of Authors:	Stelten, Stephanie; Amsterdam UMC - Locatie VUMC, Epidemiology and Biostatistics<br>Hoedjes, Meeke ; Tilburg University, Medical and Clinical Psychology<br>Kenter, Gemma; Amsterdam UMC - Locatie AMC, Obstetrics and Gynaecology; Amsterdam UMC - Locatie VUMC, Obstetrics and Gynaecology<br>Kampman, Ellen; Wageningen University and Research, Division of Human Nutrition and Health<br>Huijsmans, Rosalie; Amsterdam UMC - Locatie VUMC, Rehabilitation Medicine<br>van Lonkhuijzen, Luc; Amsterdam UMC - Locatie AMC, Obstetrics and Gynaecology<br>Buffart, LM; Radboudumc, Physiology
**Primary Subject Heading**:	Oncology
Secondary Subject Heading:	Nutrition and metabolism, Sports and exercise medicine
Keywords:	Nutritional support < ONCOLOGY, Gynaecological oncology < ONCOLOGY, NUTRITION & DIETETICS, SPORTS MEDICINE, REHABILITATION MEDICINE, Epidemiology < ONCOLOGY

SCHOLARONE™ Manuscripts

I, the Submitting Author has the right to grant and does grant on behalf of all authors of the Work (as defined in the below author licence), an exclusive licence and/or a non-exclusive licence for contributions from authors who are: i) UK Crown employees; ii) where BMJ has agreed a CC-BY licence shall apply, and/or iii) in accordance with the terms applicable for US Federal Government officers or employees acting as part of their official duties; on a worldwide, perpetual, irrevocable, royalty-free basis to BMJ Publishing Group Ltd ("BMJ") its licensees and where the relevant Journal is co-owned by BMJ to the co-owners of the Journal, to publish the Work in this journal and any other BMJ products and to exploit all rights, as set out in our licence.

The Submitting Author accepts and understands that any supply made under these terms is made by BMJ to the Submitting Author unless you are acting as an employee on behalf of your employer or a postgraduate student of an affiliated institution which is paying any applicable article publishing charge ("APC") for Open Access articles. Where the Submitting Author wishes to make the Work available on an Open Access basis (and intends to pay the relevant APC), the terms of reuse of such Open Access shall be governed by a Creative Commons licence – details of these licences and which Creative Commons licence will apply to this Work are set out in our licence referred to above.

Other than as permitted in any relevant BMJ Author's Self Archiving Policies, I confirm this Work has not been accepted for publication elsewhere, is not being considered for publication elsewhere and does not duplicate material already published. I confirm all authors consent to publication of this Work and authorise the granting of this licence.

For peer review only

Rationale and study protocol of the Physical Activity and Dietary intervention in women with OVArian cancer (PADOVA) study: A randomised controlled trial to evaluate effectiveness of a tailored exercise and dietary intervention on body composition, physical function and fatigue in women with ovarian cancer undergoing chemotherapy

S. Stelten[1], M. Hoedjes[2], G.G. Kenter[3,4,5], E. Kampman[6], R.J. Huijsmans[7], L.R.C.W. van Lonkhuijzen[4], L.M. Buffart[8]

Correspondence

Laurien M. Buffart

Geert Grooteplein Zuid 10

6500 HB Nijmegen, the Netherlands

E-mail address: laurien.buffart@radboudumc.nl

Tel: +31 20 444 9931

Authors affiliations

[1]Amsterdam UMC, Vrije Universiteit Amsterdam, Department of Epidemiology and Biostatistics, Amsterdam Public Health research institute, Cancer Center Amsterdam, de Boelelaan 1089a, 1081 HV Amsterdam, Netherlands

[2]Tilburg University, Center of Research on Psychology in Somatic diseases, Department of Medical and Clinical Psychology, Warandelaan 2, 5037 AB Tilburg, Netherlands

[3]Amsterdam UMC, Vrije Universiteit Amsterdam, Department of Obstetrics and Gynaecology, Cancer Center Amsterdam, Center for Gynaecologic Oncology Amsterdam (CGOA), de Boelelaan 1117, 1081 HV Amsterdam, Netherlands

[4]Amsterdam UMC, Univ(ersity) of Amsterdam, Department of Obstetrics and Gynaecology, Center for Gynaecologic Oncology Amsterdam (CGOA), Meibergdreef 9, 1105 AZ Amsterdam, Netherlands

[5]The Netherlands Cancer Institute – Antoni van Leeuwenhoek Hospital, Department of Gynaecology, Center for Gynaecologic Oncology Amsterdam (CGOA), Plesmanlaan 121, 1066 CX Amsterdam, Netherlands

[6]Wageningen University and Research, Division of Human Nutrition and Health, P.O. Box 17, 6700 AA Wageningen, Netherlands

[7]Amsterdam UMC, Vrije Universiteit van Amsterdam, Department of Rehabilitation Medicine, de

For peer review only

Boelelaan 1117, 1081HV Amsterdam, Netherlands

[8]Radboud University Medical Center, Department of Physiology, Radboud Institute of Health Sciences,

P.O. Box 9101, 6500 HB Nijmegen, Netherlands

Word count: 3986 (excluding title page, abstract, references, figures and tables)

For peer review only

For peer review only - http://bmjopen.bmj.com/site/about/guidelines.xhtml

ABSTRACT

Introduction: As a consequence of ovarian cancer and its treatment, many women with ovarian cancer have to deal with reduced physical function, fatigue and loss of weight and/or muscle mass, compromising quality of life. Exercise and dietary interventions can positively influence body composition, physical fitness and function and fatigue in patients with cancer. However, there is no data from randomised controlled trials on the effectiveness of exercise and dietary interventions in patients with ovarian cancer. Due to a complex disease trajectory, a relatively poor survival and distinct disease- and treatment-induced side effects, it is unclear whether exercise and dietary interventions that were shown feasible and effective in other types of cancer produce comparable results in patients with ovarian cancer. The aim of this paper is to present the design of the multicenter randomised controlled Physical Activity and Dietary intervention in OVArian cancer (PADOVA) trial and to describe how the exercise and dietary intervention is tailored to specific comorbidities, and disease- and treatment induced adverse effects in patients with ovarian cancer.

Methods and analysis: Adult women with primary epithelial ovarian cancer who are scheduled to undergo first-line (neo)adjuvant chemotherapy (n=122) are randomly allocated to a combined exercise and dietary intervention or a usual care control group during chemotherapy. Primary outcomes are body composition, physical function and fatigue. Outcome measures will be assessed before the start of chemotherapy, three weeks after completion of chemotherapy and 12 weeks later. The exercise and dietary intervention was tailored to ovarian cancer specific comorbidities and adverse effects of ovarian cancer and its treatment following the I3-S strategy.

Ethics and dissemination: This study has been approved by the medical ethical committee of the Amsterdam UMC (reference: 018). Results of the study will be published in international peer-reviewed journals.

Trial registration number: Netherlands Trial register (NTR6300)

Strengths and limitations of this study

- This is a randomised controlled trial in women with ovarian cancer.
- Systematic development and tailoring of the exercise and dietary intervention will improve compliance to the intervention and prevent drop out.

For peer review only - http://bmjopen.bmj.com/site/about/guidelines.xhtml

- This study will significantly contribute to the scientific evidence on the benefits of exercise and dietary support during chemotherapy in an understudied group of patients.
- This study offers a combined exercise and dietary intervention and therefore the effects of an exercise or dietary intervention alone cannot be disentangled.

INTRODUCTION

Ovarian cancer is the seventh most common type of cancer worldwide, with 239.000 new cases annually.[1] Ovarian cancer is often diagnosed at an advanced International Federation of Gynaecology and Obstetrics (FIGO) stage resulting in a poor overall prognosis.[2] Overall 5-year survival rate for ovarian cancer is 30-40%, but ranges from 92% in patients with FIGO stage I at diagnosis to 29% in patients with FIGO stage IV.[2]

The majority (90%) of malignant ovarian tumors are of epithelial origin.[2] Standard care for epithelial ovarian cancer includes cytoreductive surgery and platinum- and taxane-based (neo)adjuvant chemotherapy.[3,4] As a consequence of ovarian cancer and its treatment, many women have physical and/or psychosocial problems such as reduced physical function and fatigue, compromising quality of life.[5-14] Additionally, previous studies reported that half of the patients suffer from sarcopenia (i.e. loss of skeletal muscle mass) or malnutrition at diagnosis and that the prevalence increased during neoadjuvant chemotherapy.[9,10,15,16] Independent from the presence of sarcopenia and malnutrition, studies reported that 24-57% of patients with ovarian cancer are overweight and 10-35% are obese.[9,10,17-21]

Observational studies in women with ovarian cancer found that sarcopenia, overweight and obesity at diagnosis, as well as loss of body weight and muscle mass during treatment, and underweight after treatment are associated with a lower survival rate.[9-11,17-22] Furthermore, observational studies among patients with cancer, not including ovarian cancer, have shown that higher levels of physical activity or physical fitness are associated with better survival.[23,24] Therefore, it may be important to prevent weight gain or involuntary weight loss, and maintain physical fitness and muscle mass during treatment.

Exercise and dietary interventions are both non-pharmacological interventions that can positively influence body composition, physical fitness and function and reduce fatigue in patients with cancer.[25-29] A meta-analysis of intervention studies among the general population[30] as well as the International

For peer review only - http://bmjopen.bmj.com/site/about/guidelines.xhtml

Society of Sports Nutrition[31] highlight that a combined exercise and dietary intervention is more effective for changing body composition than an exercise or dietary intervention alone. Most previous studies examining exercise or dietary interventions in patients with cancer were conducted in patients with breast cancer. It is unclear whether the effects of exercise and dietary interventions found in patients with breast cancer can be generalized to women with ovarian cancer. Compared with breast cancer, ovarian cancer is often detected in a more advanced stage[32] and in older women. Ovarian cancer also has a substantially different treatment trajectory, i.e. different type of chemotherapy and other adjuvant therapy regimens. A few previous pilot studies have indicated that low-to-moderate intensity exercise interventions[33,34] or a combined exercise and dietary intervention[35] during chemotherapy are feasible in women with ovarian cancer. Adequately powered randomised controlled trials (RCT) evaluating the effect of an exercise and dietary intervention during treatment in patients with ovarian cancer are lacking. Therefore, the Physical Activity and Dietary intervention in OVArian cancer (PADOVA) study was initiated. The PADOVA study aims to evaluate the effectiveness of a combined exercise and dietary intervention during chemotherapy on body composition, physical function, and fatigue as primary outcomes, compared to an usual care control group in women undergoing chemotherapy for ovarian cancer. Secondary outcomes are physical activity and fitness, dietary intake, Body Mass Index (BMI), patients reported outcomes and treatment toxicity and completion rates. The secondary aim is to conduct an extensive process evaluation to examine how and why the intervention is (in)effective.

To optimize intervention feasibility and study retention rates, we aim to offer exercise and dietary interventions that are specifically tailored to the comorbidities, disease- and treatment induced adverse effects that individual patients with ovarian cancer may face. This paper presents the design of the multicenter randomised controlled PADOVA trial, and describes how the combined exercise and dietary intervention can be tailored specifically to patients with ovarian cancer.

METHODS AND ANALYSIS

The PADOVA study is a multicenter, single blind, randomised controlled trial. This study is funded by Dutch Cancer Society (VU 2015-7950), sponsored by Amsterdam UMC and approved by the medical ethical committee of Amsterdam UMC. Patient inclusion and data collection has started in February 2018 and is currently ongoing.

For peer review only - http://bmjopen.bmj.com/site/about/guidelines.xhtml

126 **Participants**

127 The study aims to include 122 adult (aged ≥18 years) women who are scheduled for (neo)adjuvant first-line chemotherapy treatment for primary epithelial ovarian cancer. Patients are excluded from this study when they have had a prior cancer diagnosis within 5 years, are not able to perform basic activities of daily living, have a contraindication for exercise (e.g. heart failure), have a cognitive disorder or severe emotional instability (e.g. schizophrenia, Alzheimer), are unable to read and/or write Dutch or have a life expectancy of less than 3 months.

Recruitment and randomisation

Patients are recruited from the Center of Gynaecologic Oncology Amsterdam (which is a collaboration of all gynaecological oncologists from Amsterdam UMC, Netherlands Cancer Institute – Antoni van Leeuwenhoek and affiliated peripheral hospitals) and Catharina hospital and its collaborating peripheral hospitals in the South of the Netherlands. After diagnosis and before the start of neoadjuvant chemotherapy (±2 weeks) or adjuvant chemotherapy (±4 weeks), the gynaecological oncologist informs patients about the PADOVA study during an out-patient clinic visit. Written informed consent is obtained from all patients prior to participation. After baseline measurement, participants are stratified by FIGO stage (low (I/II) versus high (III/IV stage) and chemotherapy regimen (primary surgery followed by chemotherapy versus neoadjuvant chemotherapy followed by interval debulking and adjuvant chemotherapy) and randomly allocated to either a combined exercise and dietary intervention or a usual care group. An independent researcher performs the randomisation by using a table of random numbers in blocks of four generated by an independent statistician. Allocation sequence is concealed from the research and clinical staff. After randomisation, patients in both the intervention and the control group receive a brochure with general information on physical activity, diet and body weight recommendations for cancer survivors.[36] These recommendations are not individualized, nor supervised. Patients who do not wish to participate in the study are invited to complete a single questionnaire examining relevant characteristics and reasons for declining participation to verify representativeness of the study population. See Figure 1 for an overview of the study design and procedures.

Development and description of the combined exercise and dietary intervention

The aims of the exercise and dietary intervention are to maintain physical fitness and function, to prevent

For peer review only - http://bmjopen.bmj.com/site/about/guidelines.xhtml

the loss of lean body mass and to maintain a healthy body weight during (neo)adjuvant chemotherapy. The intervention starts at the first cycle of chemotherapy and continues until three weeks after the last cycle. In general, patients receive six cycles of chemotherapy (duration of ±18 weeks). In case of dose delay or discontinuation of chemotherapy treatment, duration of the intervention and range of time between study measurements might differ between patients. For optimal intervention feasibility and study retention rates it is important to offer an exercise and dietary intervention in the PADOVA study which is specifically tailored to patients with ovarian cancer. The exercise intervention was based on the exercise intervention that has previously been shown to effectively maintain physical fitness, limit fatigue and enhance quality of life during chemotherapy in patients with breast cancer.[28] The dietary intervention was based on Dutch and international guidelines on general nutritional support for patients with cancer and nutritional support for malnourished patients or patients with ovarian cancer.[37-41] These interventions were tailored via the I3-s strategy to ovarian cancer specific comorbidities, adverse effects of ovarian cancer (e.g. ascites) and its surgical and chemotherapy treatment. The I3-S strategy was introduced in 2015 by Dekker et al.[42] to develop comorbidity-related adaptations to exercise therapy, and has previously been used to tailor exercise interventions to potential comorbidities and adverse effects of breast cancer treatment[43] and patients with knee osteoarthritis.[44]

The I3-S strategy consists of four steps, via which relevant information on the specific disease is collected. In the first step, information on comorbidities that occur in patients with ovarian cancer was gathered. All registered comorbidities of patients (n=109) who were treated for ovarian cancer in 2016 in the Amsterdam UMC were collected from patients records. Comorbidities were categorized according to International Classification of Diseases, 10th revision[45] (see Table 1 for an overview of all comorbidities).

For peer review only

For peer review only - http://bmjopen.bmj.com/site/about/guidelines.xhtml

179 *Table 1: Comorbidities in patients with ovarian cancer (N=109)*

**Comorbidity**	**%**
Hypertensive diseases (i.e. hypertension)	28
Ischaemic heart diseases (i.e. angina pectoris, myocardial infarction (>6 months), percutaneous transluminal coronary angioplasty, coronary artery bypass grafting)	6
Other forms of heart disease (i.e. atrial fibrillation and flutter, other cardiac arrhythmias, heart failure, other forms of heart disease not specified)	9
Cerebrovascular diseases (i.e. stroke, not specified as haemorrhage or infarction)	4
Diseases of arteries, arterioles and capillaries (i.e. aortic aneurysm, peripheral vascular diseases)	3
Diseases of veins, lymphatic vessels and lymph nodes, not elsewhere classified (i.e. phlebitis and thrombophlebitis)	1
Chronic lower respiratory diseases (i.e. chronic obstructive pulmonary disease, asthma)	16
Diabetes Mellitus (unspecified)	8
Disorders of thyroid gland (i.e. hypothyroidism/hyperthyroidism)	6
Disorders of other endocrine glands (i.e. hypoparathyroidism/hyperparathyroidism)	1
Episodic and paroxysmal disorders (i.e. transient ischaemic attack)	1
Other disorders of the nervous system (unspecified) 1	1
Dementia in other diseases classified elsewhere (i.e. dementia in Parkinson disease)	1
Diseases of liver (unspecified)	1
Disorders of gallbladder, biliary tract and pancreas (i.e. cholelithiasis)	5
Renal failure (unspecified)	1
Soft tissue disorders (i.e. rheumatism, unspecified)	2
Tuberculosis	1
Malignant neoplasms (excl. basal cell carcinoma)	7

For peer review only - http://bmjopen.bmj.com/site/about/guidelines.xhtml

Comorbidities with a prevalence of ≥5% were included in steps 2-4 of the I3-S strategy. In addition to comorbidities, the potential adverse effects of ovarian cancer and its treatment were added to the inventory. These adverse effects were derived from the literature[46], guidelines[47,48] and expert meetings with (gynaecologic) oncologists. We included the adverse effects (incidence ≥1%) of carboplatin and paclitaxel as these are currently standard chemotherapy treatments of ovarian cancer.[4] In addition, we included all potential adverse effects of surgery and ovarian cancer itself. We focused on potential adverse effects relevant for health-care providers delivering the exercise or dietary interventions. As a consequence, we excluded descriptions of acute adverse effects of chemotherapy monitored by treating physicians during admission to the hospital. Additionally, we checked overlap of comorbidities and adverse effects of ovarian cancer and its treatment with previously published I3-S papers for knee osteoarthritis[44] and breast cancer[43] or nutritional guidelines.[37-39,41] The following comorbidities (i.e. hypertensive diseases, ischaemic heart diseases, other forms of heart disease, chronic lower respiratory diseases, Diabetes Mellitus), and adverse effects (clinical parameters such as leukopenia/neutropenia, trombopenia, anemia; and symptoms of dyspnea, nausea, vomiting or diarrhea, skin and nail changes, fever, dizziness, decreased or increased heart rate, change in body weight, depression, numbness/loss of sensation, hearing and/or visual impairments, fatigue, pain and chest pain) were described in previous publications[43,44] or nutritional guidelines[37-39,41] and are not presented in this paper.

In the second and third step, contraindications and restrictions on exercise training were gathered as well as solutions for exercise training in ovarian cancer specific comorbidities, adverse effects of ovarian cancer and its treatment. This was based on literature[42-44,49], guidelines[38] and/or expert opinions of (gynaecologic) oncologists and physical therapists specialized in oncology.

In the final step, comorbidities, adverse effects of ovarian cancer and its treatment, but also exercise contraindications and restrictions were translated to clinical parameters and symptoms that can be monitored during the intervention of the PADOVA study. All information was synthesized in a framework (Table 2).

For peer review only - http://bmjopen.bmj.com/site/about/guidelines.xhtml

Table 2: framework with alternations for the exercise and dietary intervention

**Comorbidities, adverse effects ovarian cancer or treatment**	**Considerations**	**Actions/strategy**
Comorbidities		
Disorders of thyroid gland (i.e. hypothyroidism/hyperthyroidism)	Consider the following complications of disorders of thyroid gland:<br>• Weight loss or weight gain	• Refer to dietitian when weight loss/-gain occurs
	• Bradycardia in hypothyroidism or tachycardia in hyperthyroidism	• Explain to patient brady-/tachycardia due to hypo-/hyperthyroidism<br>• Monitor symptoms: in case of persistent co-existing symptoms (dyspnea, anxiety, fatigue): terminate exercise and refer to physician
	• Low energy/fatigue in hypothyroidism	• Explain to patient fatigue due to hypothyroidism<br>• Refer to physician when fatigue does not reduce in a few weeks
Disorders of gallbladder, biliary tract and pancreas (i.e. cholelithiasis)		• Monitor symptoms and in case of persistent pain: terminate exercise and refer to physician
Malignant neoplasms (excl. basal cell carcinoma) [1]	Consider comorbidities and adverse effects of the malignant neoplasm that might interfere with the intervention	
Adverse effects ovarian cancer		
Ascites (60%)	Consider the following complications of ascites:<br>• Discomfort	• Adjust exercise to a comfortable intensity or posture
	• Pleural effusion and/or shortness in breath	• In case of disproportional dyspnea: terminate exercise session and refer to physician
	• Diminished nutritional intake	• Refer to dietitian
Adverse effects of chemotherapy and surgery		
Abdominal wound		• In the post-operative period (4-6 weeks) exercise is allowed, but pressure on the abdomen should be avoided therefore the 'abdominal crunch' and 'pullover' could be replaced with a 'lateral raise' and 'leg extension'<br>• After 4-6 weeks isometric exercises could be replaced by eccentric exercises<br>• Monitor symptoms: in case of pain or discomfort decrease training intensity or resistance
	Consider the following complications of an abdominal wound:	

For peer review only

For peer review only - http://bmjopen.bmj.com/site/about/guidelines.xhtml

	• Fever >38.5 degrees Celsius due to wound infection	• Contra-indication for exercise, refer to physician
Intestinal stoma	Consider the following complications of an intestinal stoma<br>• High output stoma (production of >1 liter per 24 hour during ≥ 3 days)	• See recommendation under 'abdominal wound'<br>• Avoid contact sport (e.g. football or martial arts)<br>• Contra-indication for exercise, refer to physician and/or dietitian
(Risk of) lymphedema in legs	Consider the following complications of lymphedema in legs:<br>• Numbness/loss of sensation	• Monitor leg volume during exercise program; ask for symptoms of lymphedema<br>• Refer to lymphedema specialist when lymphedema is present, or when leg volumes increases and symptoms arise (advice on how to progress with exercise)<br>• Be careful with exercises that include walking, running or balance to prevent falls<br>• Advise patient to wear good fitting, stable footwear with good grip under surface
Deep vein thrombosis (in leg)		• Contra-indication for exercise, refer to a physician when the following symptoms occur: pain in the leg, red or discolored skin, or a feeling of warmth
Thrombophlebitis		• Avoid pressure or impact on affected area<br>• Monitor symptoms and in case of pain or discomfort decrease training intensity or resistance
Nervousness/confusion	Consider the following cause of nervousness/confusion<br>• Sever anxiety or a psychiatric disorder	• Give the patient time to discuss feelings or thoughts<br>• Contra-indication for exercise, refer to a physician when a serious psychiatric disorder might be present
Gastro-intestinal symptoms (i.e. anorexia, dyspepsia, constipation, taste disorder, dry mouth, mouth ulcers, stomatitis)	Consider the following complications of gastro-intestinal symptoms:<br>• Diminished nutritional intake	• Refer to dietitian and/or physician
Melaena		• Contra-indication for exercise, refer to a physician

209 *[1] Patients with a current other malignancy or prior diagnosis (within 5 years) of cancer were excluded from participation in the PADOVA study*

For peer review only - http://bmjopen.bmj.com/site/about/guidelines.xhtml

Exercise intervention

The exercise intervention consists of two one-hour exercise sessions per week including moderate-to-high intensity resistance and aerobic exercises. The exercise sessions are supervised by a physical therapist specifically trained in treating oncology patients, to inform patients on and monitor appropriate and safe exercise strategies. These physical therapists are affiliated with a nation-wide network that includes >600 physical therapy practices. This enables to offer the intervention close to a patients' home. Depending on physical therapy practice, patients train in small groups with other (non-)PADOVA patients (with cancer).

The exercise intervention is based on the exercise training principles (i.e. specificity, progression, overload, initial values, reversibility and diminishing returns).[50] The sessions start with a warming up of 10 minutes. Resistance exercises targeting six large muscle groups are conducted for 20 minutes per session. Prescribed exercises include vertical row, leg press, bench press, pull over, abdominal crunch and lunge. Due to the abdominal wound in the post-operative period (4-6 weeks) eccentric exercises with the abdominal muscles and pressure on the abdomen are prevented by omitting heavy lifting and exercises such as abdominal crunch and pullover. Instead, exercises with an isometric use of the abdominal muscles such as lateral raise or leg extension, are performed. An overview of all adaptations for patients with ovarian cancer is shown in Table 2. One repetition maximum (1RM) testing is repeated every three weeks, in line with chemotherapy regimen, to ensure adequate training intensity over time. Load of each resistance exercise is 70-80% of the 1RM with a gradual increase per week in between 1RM testing. Exercises are performed in 2 sets of 8-10 repetitions. When the participant is unable to perform 2 sets of 10 repetitions, or when the Borg Scale of perceived exertion exceeds 15, load will be decreased by one step. When the Borg Scale of perceived exertion decreases to <12, the load will be increased. Aerobic exercises are conducted for 30 minutes per session, with an intensity of 50-80% (gradually increasing) of the maximal work load as estimated by the steep ramp test[51]. This test is repeated every 6 weeks to ensure adequate work load over time. When the Borg Scale of perceived exertion decreases to a score of ≤12 or increases to ≥16 the work load is adjusted.[52] In addition to the supervised sessions, patients are encouraged to be physically active on at least three additional days a week for 30 minutes to meet the recommended physical activity levels.[40]

Dietary intervention

The dietary intervention is provided by oncology dietitians once every three weeks during face-to-face

For peer review only - http://bmjopen.bmj.com/site/about/guidelines.xhtml

sessions of 30-45 minutes each at the hospital or by telephone using motivational interviewing techniques.[53] Motivational interviewing is an effective counselling method for achieving health behaviour change using techniques as reflective listening and summarizing, focusing on what the patient wants, thinks and feels.[54] The PADOVA dietary intervention is based on Bandura's Social Cognitive theory (SCT).[55] Lifestyle interventions based on SCT have been shown to improve health behaviours in patients during and after cancer treatment,[56] and generally focus on improving self-efficacy, dealing with sociostructural factors (impediments/barriers and facilitators), managing outcome expectations, and setting goals to improve health behaviours.[55] The Behavior Change Techniques (BCT's) used to promote heal behaviour change are defined according to the BCT Taxonomy version v1 [57] and listed in supplementary table A. Content of the dietary intervention is based on the dietary guideline set by the World Cancer Research Fund (WCRF)/American Institute for Cancer Research (AICR)[40] and an sufficient total protein intake of at least 1.2 grams of protein per kg body weight per day[41,58] and 25 grams of protein per meal, since such evenly distributed protein intake is expected to optimize muscle protein synthesis.[59] Counselling is tailored to the nutritional needs of each individual patient according to body composition, nutritional status and dietary intake during chemotherapy. Patients who are (at risk of) malnutrition are primarily counselled for prevention of weight loss by maintaining sufficient caloric intake, particularly protein intake. Patients who are not at risk of developing malnutrition are primarily counselled to meet the dietary guidelines set by the WCRF/AICR. During each counselling session, patients will receive feedback on their body weight, BMI, body composition assessed via bio-electric impedance analysis (BIA), diet quality, and on the extent to which they meet the protein goals and WCRF/AICR dietary recommendations. Extended information on the content per dietary counselling session is provided in supplementary table B.

Control group

Women in the control group will receive usual care during chemotherapy, which includes referral to a dietitian when malnutrition is detected by the gynaecological oncologist. Usual care does not include structured exercise and/or dietary counselling. In order to prevent nonparticipation and drop-out, patients in the control group are offered a maximum of three exercise and three dietary counselling sessions in twelve weeks after completion of chemotherapy and the first follow up measurement.

For peer review only

Outcome measurements

An investigator blinded from group allocation conducts measurements at three time points. Participants are instructed not to reveal their group allocation. Baseline measurements are conducted before randomisation and the start of chemotherapy (T0), the second measurement three weeks after completion of chemotherapy (T1) and the last measurement (T2) twelve weeks after T1. An overview of all outcome measurements is presented in Table 3.

Table 3: Summary of outcome measurements

Outcome	Instrument	T0	T1	T2
Primary outcomes				
Body composition	Computed tomography imaging for the assessment of skeletal muscle area	X	X	
	Bio-electric impedance analysis for the assessment of fat mass	X	X	X
Physical fatigue	Multidimensional Fatigue Inventory[60]	X	X	X
Physical function	European Organisation Research and Treatment of Cancer-Quality of life questionnaire-Core 30[61]	X	X	X
Secondary outcomes				
Physical activity	Accelerometer (Actigraph) for objective measurement of physical activity in seven consecutive days during all waking hours	X	X	X
	Physical Activity Scale for the Elderly[62] for self-reported levels of physical activity	X	X	X
Physical fitness	Maximum oxygen uptake (peak VO_2) during a maximum exercise test on a cycle ergometer using a ramp protocol for the assessment of cardiorespiratory fitness	X	X	X
	Hand held dynamometer for the assessment of muscle strength	X	X	X
Body Mass Index	Body height on a calibrated scale to the nearest 0.1 mm	X	X	X
	Body weight on a calibrated scale to the nearest 0.1 kg	X	X	X
Dietary intake; WCRF/AICR guidelines	Food frequency questionnaire (developed by Wageningen University)	X	X	X
Dietary intake; protein intake	Self-composed food frequency questionnaire	X	X	X
Health-related quality of life and symptoms	European Organisation Research and Treatment of Cancer-Quality of life questionnaire – Ovarian cancer (OV28)[63]	X	X	X
	European Organisation Research and Treatment of Cancer-Quality of life questionnaire - Chemotherapy-induced peripheral neuropathy (CIPN20)[64]	X	X	X
	Hospital Anxiety and Depression Scale[65]	X	X	X
	Pittsburgh Sleep Quality Index[66]	X	X	X
Chemotherapy therapy completion rates and treatment toxicity	Medical records of which chemotherapy completion rates will be assesses as the relative dose intensity, i.e., the amount of particular		X	

For peer review only - http://bmjopen.bmj.com/site/about/guidelines.xhtml

	chemotherapy given in relation to the originally planned chemotherapy dose.			
Other study parameters				
Blood sampling	Venous blood sample (40 ml)	X	X	
Smoking and sociodemographics	Self-composed questionnaire	X		
Contamination of control group[1]	Self-composed questionnaire		X	

[1] Parameter will only be assessed in the control group

Primary outcomes

Primary outcomes are body composition, physical function and fatigue. Body composition is assessed via skeletal muscle area and fat mass. Skeletal muscle area is assessed at T0 and T1 using routine computed tomography (CT) imaging (first image extending from the third lumbar vertebra to iliac crest) conducted for diagnostic purposes. CT is considered the gold standard for assessment of muscle mass in cancer patients.[67] Because the CT of the third lumbar vertebra is not yet validated for the assessment of body fat mass,[68] fat mass is assessed with a non-invasive measurement BIA.[69] Physical function is assessed using the physical function subscale of the validated European Organisation Research and Treatment of Cancer-Quality of life questionnaire-Core 30.[61] Physical fatigue is assessed with the physical fatigue subscale of the Multidimensional Fatigue Inventory.[60]

Secondary outcomes

Secondary outcomes are physical activity, physical fitness (i.e. cardiorespiratory fitness and muscle strength), dietary intake, BMI, health-related quality of life and symptoms of neuropathy, anxiety and depression, sleep disturbances, chemotherapy treatment toxicity and completion rates. The measurement instruments for the assessment of the secondary outcomes are presented in Table 3.

Other study parameters

During the T0 and T1 visit, a venous blood sample is drawn and stored in the PADOVA biobank for future biomarker studies (e.g. to assess immune system functioning). Because collection of blood samples is an addition to participation in the PADOVA study, an additional informed consent is obtained. Covariates such as clinical data (e.g. cancer subtype, FIGO stage) and sociodemographic characteristics (e.g. age, ethnicity) are assessed at baseline. Contamination (i.e. received supervised exercise and/or dietary counselling) of the control group is assessed by a questionnaire at T1.

For peer review only

For peer review only - http://bmjopen.bmj.com/site/about/guidelines.xhtml

Process evaluation

A process evaluation is conducted using a mixed-method approach (using both quantitative and qualitative research methods). Quantitative data on behavioral counselling skills of dietitians is assessed during counselling sessions with the Behavior Change Counselling Inventory (BECCI).[70] Patients in the intervention group fill out the Health Care Climate Questionnaire (HCCQ) to assess the extent to which they perceive an autonomy supportive environment during counseling.[71] Quantitative data on behavioral determinants of physical activity and dietary behavior (i.e. outcome expectations, self-efficacy for eating and exercise habits, sociostructural factors, stage of change, type of motivation and knowledge) are assessed on T0, T1 and T2 by questionnaires in all patients. The following process evaluation components are examined by physical therapists and dietitians and reported in a form: dose delivered, dose received and fidelity. Acceptability of the intervention is assessed by a questionnaire filled out by patients and semi-structured interviews among patients, physical therapists and dietitians conducted by a researcher. Interviews will be transcribed verbatim, coded in several phases[72,73] and analyzed with Atlast.ti. Analyses are partly performed concurrently with data collection because interviews will be held until data saturation is reached. An overview of all measurement instruments for the process evaluation is presented in Table 4.

Table 4: Overview of all measurement instruments for the process evaluation

Outcome	Instrument	T0	T1	T2
Process evaluation				
Behavioral determinants	Multidimensional Outcome Expectations for Exercise Scale[62]	X	X	X
	Self-efficacy for eating and exercise habits – self-composed items	X	X	X
	Sociostructural factors, self-composed items	X	X	X
	Stage of change[74]	X	X	X
	Type of motivation: Treatment Self-Regulation Questionnaire[75]	X	X	X
	Knowledge (on WCRF recommendations): Items used in previous study[76]	X	X	X
Acceptability of intervention	Self-composed items[1]		X	
	The Health Care Climate Questionnaire – short form (6 items)[71]		X	
Acceptability of intervention	Semi-structured interviews in healthcare professionals[2]		X[1]	X[4]
	Semi-structured interviews in participants		X[1]	X[4]

For peer review only - http://bmjopen.bmj.com/site/about/guidelines.xhtml

Dose delivered	Checklist[2] – Amount of (components of) sessions provided by physical therapist/dietitian	Daily, during intervention
Dose received	Checklist[2] – Amount of (components of) sessions participants actively engaged in	Daily, during intervention
Fidelity	Checklist[2] – Extend to which the intervention was executed as prescribed in the protocol (e.g. reasons for and amount of adaptations to the protocol)	Daily, during intervention
Behavioral counselling skills[3]	Behavior Change Counselling Inventory[70]	During intervention

[1] Parameters will only be assessed in intervention group

[2] Parameter will be examined in physical therapists and dietitians

[3] Parameter will be examined in dietitians

[4] Parameter will only be assessed in the control group

Sample size calculation

Sample size calculation is based on the results of a previous RCT among patients with breast cancer that evaluated the effects of a combined aerobic and resistance exercise intervention (similar to the PADOVA study),[28] a pilot study among patients with ovarian cancer that evaluated the feasibility of an exercise intervention[34] and on clinically relevant differences in body composition and peak oxygen uptake.[34,77-82] With 53 patients per study-arm, we are able to detect a clinically relevant between group difference in effects directly post-intervention on physical function (10 points), physical fatigue (2.7 points), body composition (3% in percentage body fat and muscle mass), and 10-15% difference in peak oxygen uptake (alpha=0.05; power= 0.80). Taking into account a dropout of 15%, we aim to include 61 patients per group. Dropout rates are based on the 10-15% dropout rates from previously conducted Dutch exercise trials in patients with cancer. [27,28,83]

Statistical analysis

The primary analysis focuses on the effects of the intervention directly after completion of chemotherapy (T1), since both the intervention and control group have received counselling from a physical therapist and/or dietitian at follow-up (T2). Data will be analysed according to the intention-to-treat principle. Intervention effects (at T1) will be assessed using linear regression analysis for continuous outcomes, by regressing the intervention on post-test value (T1) of the outcome, adjusting for baseline values (T0). To examine whether intervention effects on body composition, physical function or fatigue are mediated by changes in physical activity and fitness, and/or diet, a series of regression analysis according to the

For peer review only - http://bmjopen.bmj.com/site/about/guidelines.xhtml

346 product-of-coefficients test will be conducted.[84] Potential effect modification by relevant demographic (e.g. age) and clinical (e.g. cancer stage, treatment regimen) variables will be explored by adding the variable and its interaction term with the intervention into the regression model.

DISCUSSION

351 This paper presents the rationale and design of the PADOVA study and the procedure of tailoring an exercise and dietary intervention to patients with ovarian cancer. The PADOVA study aims to examine the effectiveness of a combined exercise and dietary intervention on body composition, physical function and fatigue in patients with ovarian cancer during chemotherapy. Additionally, an process evaluation is conducted to examine the effective and ineffective components of the PADOVA intervention.

A strength of this study is its randomized controlled design in women with ovarian cancer and systematic development of the exercise and dietary intervention by the I3-S strategy to ensure adequate tailoring of the intervention to this patient specific group. Because the intervention is adjusted to comorbidities, disease- and treatment induced effects, we expect compliance to the intervention to be high and drop out to be low. Another strength is the process evaluation because it retrieves information on how and why the intervention is (un)successful.[85] With this information the exercise and dietary intervention will be improved before the combined intervention is implemented in clinical practice.

Aiming to maximize benefits on body composition, we combined an exercise and dietary intervention. Consequently, we are unable to disentangle the effects of the exercise and dietary intervention. However, we plan to perform a mediation analysis to explore whether the intervention effects on body composition can be explained by changes in exercise or dietary components. Another limitation of this study is its inability to study long-term effects of the intervention compared to the control group. In the PADOVA study, exercise and dietary counselling will be offered to the control group after the first follow up measurement in order to prevent nonparticipation and dropout and to limit contamination (exercise and dietary intervention) in the control group during chemotherapy.

Close collaboration with physical therapists specifically educated to supervise patients with cancer, affiliated with a network throughout the Netherlands has the advantage that the exercise intervention can be offered close to a patients' home. This increases the reach and scalability of the intervention. Consequentially, this might also lead to small variations in the implementation of the intervention protocol by each individual physical therapist.

For peer review only - http://bmjopen.bmj.com/site/about/guidelines.xhtml

This study will contribute to the evidence on the potential benefits of an exercise and dietary intervention in patients with ovarian cancer during chemotherapy treatment who often face a complex and unfavorable disease trajectory. If proven effective, a combined exercise and dietary intervention for patients with ovarian cancer can be implemented in clinical practice.

Patient and public involvement

Patients were involved in the development of study specific patient information and they were asked to assess the burden of time required to participate in this study. The opinion of patients has been considered to improve the readability of the patient information sheets on the study. During the study patients will be interviewed (e.g. on acceptability of the intervention) as part of the process evaluation. This information could be important for implementation of the intervention in clinical practice. Study results will be presented to patients in collaboration with the patient community.

Ethics and dissemination

This study has been approved by the medical ethical committee of the Amsterdam UMC (reference: 018). Additional approval was obtained for the participating hospitals. The trial is registered in the Netherlands Trial Register. Signed informed consent is required of all included participants. Results of the study will be published in international peer-reviewed journals.

Acknowledgements

Not applicable.

Contributors

EK, GGK, LMB and MH conceived the study. GGK, LMB, LRCWL, MH and SS designed the study. LMB, MH, RJH and SS designed the intervention. LMB, MH and SS wrote the manuscript. All authors read and approved the final manuscript.

Funding

The PADOVA study is funded by the Dutch Cancer Society, grant number VU 2015-7950. The Dutch Cancer Society was not involved in the design of the study, the collection, analysis and interpretation of

For peer review only - http://bmjopen.bmj.com/site/about/guidelines.xhtml

data, nor in writing the manuscript.

Competing interests

The authors declare that they have no competing interests.

Patient consent for publication

Not required.

Provenance and peer review

Not commissioned; externally peer reviewed.

Figure 1: Overview of the PADOVA study design and procedures

REFERENCES

1. Ferlay J, Soerjomataram I, Dikshit R, et al. Cancer incidence and mortality worldwide: sources, methods and major patterns in GLOBOCAN 2012. *Int J Cancer* 2015;136(5):E359-86. doi: 10.1002/ijc.29210 [published Online First: 2014/09/16]
2. Reid BM, Permuth JB, Sellers TA. Epidemiology of ovarian cancer: a review. *Cancer Biol Med* 2017;14(1):9-32.
3. Netherlands CCCt. Epithelial Ovarian Carcinoma; National guidelines 2.0. 2012
4. Group obotEGW, Raja FA, Ledermann JA, et al. Newly diagnosed and relapsed epithelial ovarian carcinoma: ESMO Clinical Practice Guidelines for diagnosis, treatment and follow-up†. *Annals of Oncology* 2013;24(suppl_6):vi24-vi32. doi: 10.1093/annonc/mdt333
5. Holzner B, Kemmler G, Meraner V, et al. Fatigue in ovarian carcinoma patients: a neglected issue? *Cancer* 2003;97(6):1564-72. doi: 10.1002/cncr.11253 [published Online First: 2003/03/11]
6. Meraner V, Gamper EM, Grahmann A, et al. Monitoring physical and psychosocial symptom trajectories in ovarian cancer patients receiving chemotherapy. *BMC Cancer* 2012;12:77. doi: 10.1186/1471-2407-12-77 [published Online First: 2012/03/01]
7. von Gruenigen VE, Huang HQ, Gil KM, et al. Assessment of factors that contribute to decreased quality of life in Gynecologic Oncology Group ovarian cancer trials. *Cancer* 2009;115(20):4857-64. doi: 10.1002/cncr.24520 [published Online First: 2009/08/20]
8. Kayl AE, Meyers CA. Side-effects of chemotherapy and quality of life in ovarian and breast cancer patients. *Curr Opin Obstet Gynecol* 2006;18(1):24-8. doi: 10.1097/01.gco.0000192996.20040.24 [published Online First: 2006/02/24]
9. Rutten IJ, van Dijk DP, Kruitwagen RF, et al. Loss of skeletal muscle during neoadjuvant chemotherapy is related to decreased survival in ovarian cancer patients. *J Cachexia Sarcopenia Muscle* 2016;7(4):458-66. doi: 10.1002/jcsm.12107 [published Online First: 2016/04/01]
10. Kumar A, Moynagh MR, Multinu F, et al. Muscle composition measured by CT scan is a measurable predictor of overall survival in advanced ovarian cancer. *Gynecol Oncol* 2016;142(2):311-6. doi: 10.1016/j.ygyno.2016.05.027
11. Mardas M, Stelmach-Mardas M, Madry R. Body weight changes in patients undergoing chemotherapy for ovarian cancer influence progression-free and overall survival. *Support Care Cancer* 2017;25(3):795-800. doi: 10.1007/s00520-016-3462-1
12. von Gruenigen VE, Frasure HE, Jenison EL, et al. Longitudinal assessment of quality of life and lifestyle in newly diagnosed ovarian cancer patients: the roles of surgery and chemotherapy. *Gynecol Oncol* 2006;103(1):120-6. doi: 10.1016/j.ygyno.2006.01.059 [published Online First: 2006/03/25]

For peer review only

For peer review only - http://bmjopen.bmj.com/site/about/guidelines.xhtml

13. Gil KM, Gibbons HE, Jenison EL, et al. Baseline characteristics influencing quality of life in women undergoing gynecologic oncology surgery. *Health Qual Life Outcomes* 2007;5:25. doi: 10.1186/1477-7525-5-25 [published Online First: 2007/05/19]
14. Wenzel L, Huang HQ, Monk BJ, et al. Quality-of-life comparisons in a randomized trial of interval secondary cytoreduction in advanced ovarian carcinoma: a Gynecologic Oncology Group study. *J Clin Oncol* 2005;23(24):5605-12. doi: 10.1200/jco.2005.08.147 [published Online First: 2005/08/20]
15. Mardas M, Jamka M, Madry R, et al. Dietary habits changes and quality of life in patients undergoing chemotherapy for epithelial ovarian cancer. *Support Care Cancer* 2015;23(4):1015-23. doi: 10.1007/s00520-014-2462-2 [published Online First: 2014/10/02]
16. Laky B, Janda M, Bauer J, et al. Malnutrition among gynaecological cancer patients. *Eur J Clin Nutr* 2007;61(5):642-6. doi: 10.1038/sj.ejcn.1602540
17. Kumar A, Bakkum-Gamez JN, Weaver AL, et al. Impact of obesity on surgical and oncologic outcomes in ovarian cancer. *Gynecol Oncol* 2014;135(1):19-24. doi: 10.1016/j.ygyno.2014.07.103 [published Online First: 2014/08/12]
18. Pavelka JC, Brown RS, Karlan BY, et al. Effect of obesity on survival in epithelial ovarian cancer. *Cancer* 2006;107(7):1520-4. doi: 10.1002/cncr.22194 [published Online First: 2006/08/31]
19. Aust S, Knogler T, Pils D, et al. Skeletal Muscle Depletion and Markers for Cancer Cachexia Are Strong Prognostic Factors in Epithelial Ovarian Cancer. *PLoS One* 2015;10(10):e0140403. doi: 10.1371/journal.pone.0140403
20. Bandera EV, Lee VS, Rodriguez-Rodriguez L, et al. Impact of Chemotherapy Dosing on Ovarian Cancer Survival According to Body Mass Index. *JAMA Oncol* 2015;1(6):737-45. doi: 10.1001/jamaoncol.2015.1796 [published Online First: 2015/07/17]
21. Kim SI, Kim HS, Kim TH, et al. Impact of underweight after treatment on prognosis of advanced-stage ovarian cancer. *J Immunol Res* 2014;2014:349546.
22. Hess LM, Barakat R, Tian C, et al. Weight change during chemotherapy as a potential prognostic factor for stage III epithelial ovarian carcinoma: a Gynecologic Oncology Group study. *Gynecol Oncol* 2007;107(2):260-5. doi: 10.1016/j.ygyno.2007.06.010 [published Online First: 2007/08/07]
23. Schmid D, Leitzmann MF. Cardiorespiratory fitness as predictor of cancer mortality: a systematic review and meta-analysis. *Annals of oncology : official journal of the European Society for Medical Oncology* 2015;26(2):272-8. doi: 10.1093/annonc/mdu250 [published Online First: 2014/07/11]
24. McTiernan A, Friedenreich CM, Katzmarzyk PT, et al. Physical Activity in Cancer Prevention and Survival: A Systematic Review. *Med Sci Sports Exerc* 2019;51(6):1252-61. doi: 10.1249/mss.0000000000001937 [published Online First: 2019/05/17]
25. Padilha CS, Marinello PC, Galvao DA, et al. Evaluation of resistance training to improve muscular strength and body composition in cancer patients undergoing neoadjuvant and adjuvant therapy: a meta-analysis. *J Cancer Surviv* 2017;11(3):339-49. doi: 10.1007/s11764-016-0592-x [published Online First: 2017/01/06]
26. Cohen CW, Fontaine KR, Arend RC, et al. A Ketogenic Diet Reduces Central Obesity and Serum Insulin in Women with Ovarian or Endometrial Cancer. *J Nutr* 2018;148(8):1253-60. doi: 10.1093/jn/nxy119 [published Online First: 2018/08/24]
27. Kampshoff CS, Chinapaw MJ, Brug J, et al. Randomized controlled trial of the effects of high intensity and low-to-moderate intensity exercise on physical fitness and fatigue in cancer survivors: results of the Resistance and Endurance exercise After ChemoTherapy (REACT) study. *BMC Med* 2015;13:275. doi: 10.1186/s12916-015-0513-2 [published Online First: 2015/10/31]
28. van Waart H, Stuiver MM, van Harten WH, et al. Effect of Low-Intensity Physical Activity and Moderate- to High-Intensity Physical Exercise During Adjuvant Chemotherapy on Physical Fitness, Fatigue, and Chemotherapy Completion Rates: Results of the PACES Randomized Clinical Trial. *J Clin Oncol* 2015;33(17):1918-27. doi: 10.1200/jco.2014.59.1081 [published Online First: 2015/04/29]
29. Demark-Wahnefried W, Morey MC, Sloane R, et al. Reach out to enhance wellness home-based diet-exercise intervention promotes reproducible and sustainable long-term improvements in health behaviors, body weight, and physical functioning in older, overweight/obese cancer survivors. *J Clin Oncol* 2012;30(19):2354-61. doi: 10.1200/jco.2011.40.0895 [published Online First: 2012/05/23]
30. Johns DJ, Hartmann-Boyce J, Jebb SA, et al. Diet or exercise interventions vs combined behavioral weight management programs: a systematic review and meta-analysis of direct comparisons. *J Acad Nutr Diet* 2014;114(10):1557-68. doi: 10.1016/j.jand.2014.07.005 [published Online First: 2014/09/27]
31. Jager R, Kerksick CM, Campbell BI, et al. International Society of Sports Nutrition Position Stand: protein and exercise. *J Int Soc Sports Nutr* 2017;14:20.

For peer review only - http://bmjopen.bmj.com/site/about/guidelines.xhtml

32. Gil KM, von Gruenigen VE. Physical activity and gynecologic cancer survivorship. *Recent Results Cancer Res* 2011;186:305-15. doi: 10.1007/978-3-642-04231-7_13 [published Online First: 2010/11/30]
33. Newton MJ, Hayes SC, Janda M, et al. Safety, feasibility and effects of an individualised walking intervention for women undergoing chemotherapy for ovarian cancer: a pilot study. *BMC Cancer* 2011;11:389. doi: 10.1186/1471-2407-11-389 [published Online First: 2011/09/09]
34. Mizrahi D, Broderick C, Friedlander M, et al. An Exercise Intervention During Chemotherapy for Women With Recurrent Ovarian Cancer: A Feasibility Study. *Int J Gynecol Cancer* 2015;25(6):985-92. doi: 10.1097/igc.0000000000000460 [published Online First: 2015/04/29]
35. von Gruenigen VE, Frasure HE, Kavanagh MB, et al. Feasibility of a lifestyle intervention for ovarian cancer patients receiving adjuvant chemotherapy. *Gynecol Oncol* 2011;122(2):328-33. doi: 10.1016/j.ygyno.2011.04.043 [published Online First: 2011/05/24]
36. Gezond leven na kanker. In: Fonds SWKO, ed.: Wereld Kanker Onderzoeks Fonds, 2017.
37. Oncoline. Algemene voedings- en dieetbehandeling 2017 [updated 01-03-2017. Available from: https://www.oncoline.nl/voedings-en-dieetbehandeling accessed 20-08-2018 2018.
38. Oncoline. Eierstokkanker 2017 [updated 01-03-2017. Available from: https://www.oncoline.nl/voeding-eierstokkanker.
39. Oncoline. Ondervoeding bij patienten met kanker 2012 [updated 15-01-2012. Available from: https://www.oncoline.nl/ondervoeding-bij-patienten-met-kanker.
40. World Cancer Research Fund / American Institute for Cancer Research. Diet, Nutrition, Physical Activity and Cancer: a Global Perspective. Continuous Update Project Expert report, 2018.
41. Arends J, Bachmann P, Baracos V, et al. ESPEN guidelines on nutrition in cancer patients. *Clin Nutr* 2016 doi: 10.1016/j.clnu.2016.07.015 [published Online First: 2016/09/18]
42. Dekker J, de Rooij M, van der Leeden M. Exercise and comorbidity: the i3-S strategy for developing comorbidity-related adaptations to exercise therapy. *Disabil Rehabil* 2016;38(9):905-9. doi: 10.3109/09638288.2015.1066451
43. van der Leeden M, Huijsmans RJ, Geleijn E, et al. Tailoring exercise interventions to comorbidities and treatment-induced adverse effects in patients with early stage breast cancer undergoing chemotherapy: a framework to support clinical decisions. *Disabil Rehabil* 2017:1-11. doi: 10.1080/09638288.2016.1260647
44. de Rooij M, van der Leeden M, Avezaat E, et al. Development of comorbidity-adapted exercise protocols for patients with knee osteoarthritis. *Clin Interv Aging* 2014;9:829-42.
45. Organization WH. International statistical classification of diseases and related health problems (10th revision) [updated 2016. 5:[Available from: https://icd.who.int/browse10/2016/en2019.
46. Farmacotherapeutisch Kompas: Zorginstituut Nederland; [Available from: https://farmacotherapeutischkompas.nl accessed 20-09-2017.
47. Oncoline. Epitheliaal ovariumcarcinoom 2.1: Integraal Kankercentrum Nederland; 2018 [updated 2018-01-15.
48. Oncoline. Ascites, versie 2.0: Integraal Kankercentrum Nederland; 2010 [updated 19-01-2010.
49. Klein I, Danzi S. Thyroid Disease and the Heart. *Circulation* 2007;116(15):1725-35. doi: doi:10.1161/CIRCULATIONAHA.106.678326
50. Campbell KL, Neil SE, Winters-Stone KM. Review of exercise studies in breast cancer survivors: attention to principles of exercise training. *British journal of sports medicine* 2012;46(13):909-16. doi: 10.1136/bjsports-2010-082719 [published Online First: 2012/09/26]
51. De Backer IC, Schep G, Hoogeveen A, et al. Exercise testing and training in a cancer rehabilitation program: the advantage of the steep ramp test. *Arch Phys Med Rehabil* 2007;88(5):610-6. doi: 10.1016/j.apmr.2007.02.013 [published Online First: 2007/05/01]
52. Borg G. Borg's perceived exertion and pain scales. Champaign, IL, US: Human Kinetics 1998.
53. Miller WR, Rollnick S. Ten things that motivational interviewing is not. *Behav Cogn Psychother* 2009;37(2):129-40. doi: 10.1017/s1352465809005128 [published Online First: 2009/04/15]
54. Miller WR. Motivational interviewing: research, practice, and puzzles. *Addict Behav* 1996;21(6):835-42. doi: 10.1016/0306-4603(96)00044-5 [published Online First: 1996/11/01]
55. A. B. Social foundations of thought and action: a social cognitive theory. NJ: Englewood Cliffs ed1986.
56. Stacey FG, James EL, Chapman K, et al. A systematic review and meta-analysis of social cognitive theory-based physical activity and/or nutrition behavior change interventions for cancer survivors. *J Cancer Surviv* 2015;9(2):305-38. doi: 10.1007/s11764-014-0413-z [published Online First: 2014/11/30]
57. Michie S, Richardson M, Johnston M, et al. The behavior change technique taxonomy (v1) of 93 hierarchically clustered techniques: building an international consensus for the reporting of behavior

For peer review only - http://bmjopen.bmj.com/site/about/guidelines.xhtml

change interventions. *Ann Behav Med* 2013;46(1):81-95. doi: 10.1007/s12160-013-9486-6 [published Online First: 2013/03/21]

58. Nitenberg G, Raynard B. Nutritional support of the cancer patient: issues and dilemmas. *Crit Rev Oncol Hematol* 2000;34(3):137-68. [published Online First: 2000/06/06]
59. Paddon-Jones D, Rasmussen BB. Dietary protein recommendations and the prevention of sarcopenia. *Curr Opin Clin Nutr Metab Care* 2009;12(1):86-90. doi: 10.1097/MCO.0b013e32831cef8b [published Online First: 2008/12/06]
60. Smets EM, Garssen B, Bonke B, et al. The Multidimensional Fatigue Inventory (MFI) psychometric qualities of an instrument to assess fatigue. *J Psychosom Res* 1995;39(3):315-25. [published Online First: 1995/04/01]
61. Aaronson NK, Ahmedzai S, Bergman B, et al. The European Organization for Research and Treatment of Cancer QLQ-C30: a quality-of-life instrument for use in international clinical trials in oncology. *J Natl Cancer Inst* 1993;85(5):365-76. [published Online First: 1993/03/03]
62. Wojcicki TR, White SM, McAuley E. Assessing outcome expectations in older adults: the multidimensional outcome expectations for exercise scale. *J Gerontol B Psychol Sci Soc Sci* 2009;64(1):33-40. doi: 10.1093/geronb/gbn032 [published Online First: 2009/02/03]
63. Greimel E, Bottomley A, Cull A, et al. An international field study of the reliability and validity of a disease-specific questionnaire module (the QLQ-OV28) in assessing the quality of life of patients with ovarian cancer. *Eur J Cancer* 2003;39(10):1402-8. [published Online First: 2003/06/27]
64. Postma TJ, Aaronson NK, Heimans JJ, et al. The development of an EORTC quality of life questionnaire to assess chemotherapy-induced peripheral neuropathy: the QLQ-CIPN20. *Eur J Cancer* 2005;41(8):1135-9. doi: 10.1016/j.ejca.2005.02.012 [published Online First: 2005/05/25]
65. Zigmond AS SR. The hospital anxiety and depression scale. *Acta Psychiatr Scand* 1983;67(6):361-70.
66. Buysse DJ, Reynolds CF, Monk TH, et al. The Pittsburgh sleep quality index: A new instrument for psychiatric practice and research. *Psychiatry Research* 1989;28(2):193-213. doi: https://doi.org/10.1016/0165-1781(89)90047-4
67. Prado CM, Birdsell LA, Baracos VE. The emerging role of computerized tomography in assessing cancer cachexia. *Curr Opin Support Palliat Care* 2009;3(4):269-75. doi: 10.1097/SPC.0b013e328331124a [published Online First: 2009/08/12]
68. Shen W, Punyanitya M, Wang Z, et al. Total body skeletal muscle and adipose tissue volumes: estimation from a single abdominal cross-sectional image. *J Appl Physiol (1985)* 2004;97(6):2333-8. doi: 10.1152/japplphysiol.00744.2004 [published Online First: 2004/08/18]
69. Bolanowski M, Nilsson BE. Assessment of human body composition using dual-energy x-ray absorptiometry and bioelectrical impedance analysis. *Medical science monitor : international medical journal of experimental and clinical research* 2001;7(5):1029-33. [published Online First: 2001/09/06]
70. Lane C, Huws-Thomas M, Hood K, et al. Measuring adaptations of motivational interviewing: the development and validation of the behavior change counseling index (BECCI). *Patient Educ Couns* 2005;56(2):166-73. doi: 10.1016/j.pec.2004.01.003 [published Online First: 2005/01/18]
71. Czajkowska Z, Wang H, Hall NC, et al. Validation of the English and French versions of the Brief Health Care Climate Questionnaire. *Health Psychol Open* 2017;4(2):2055102917730675.
72. Boeije H. Analysis in qualitative research: Sage 2009.
73. Green J, Thorogood N. Qualitative methods for health research: Sage 2013.
74. Prochaska JO, Velicer WF. The transtheoretical model of health behavior change. *Am J Health Promot* 1997;12(1):38-48. doi: 10.4278/0890-1171-12.1.38 [published Online First: 1997/08/05]
75. Levesque CS, Williams GC, Elliot D, et al. Validating the theoretical structure of the Treatment Self-Regulation Questionnaire (TSRQ) across three different health behaviors. *Health Educ Res* 2007;22(5):691-702. doi: 10.1093/her/cyl148 [published Online First: 2006/12/02]
76. Vrieling A, Visser A, Hoedjes M, et al. Increasing awareness and knowledge of lifestyle recommendations for cancer prevention in Lynch syndrome carriers: Randomized controlled trial. *Clin Genet* 2018;93(1):67-77. doi: 10.1111/cge.13076 [published Online First: 2017/06/21]
77. Demark-Wahnefried W, Peterson BL, Winer EP, et al. Changes in weight, body composition, and factors influencing energy balance among premenopausal breast cancer patients receiving adjuvant chemotherapy. *J Clin Oncol* 2001;19(9):2381-9. doi: 10.1200/jco.2001.19.9.2381 [published Online First: 2001/05/02]
78. Freedman RJ, Aziz N, Albanes D, et al. Weight and body composition changes during and after adjuvant chemotherapy in women with breast cancer. *J Clin Endocrinol Metab* 2004;89(5):2248-53. doi: 10.1210/jc.2003-031874 [published Online First: 2004/05/06]

For peer review only

79. Campbell KL, Van Patten CL, Neil SE, et al. Feasibility of a lifestyle intervention on body weight and serum biomarkers in breast cancer survivors with overweight and obesity. *J Acad Nutr Diet* 2012;112(4):559-67. doi: 10.1016/j.jada.2011.10.022 [published Online First: 2012/06/20]
80. Blauwhoff-Buskermolen S, Versteeg KS, de van der Schueren MA, et al. Loss of Muscle Mass During Chemotherapy Is Predictive for Poor Survival of Patients With Metastatic Colorectal Cancer. *J Clin Oncol* 2016;34(12):1339-44. doi: 10.1200/jco.2015.63.6043 [published Online First: 2016/02/24]
81. Battaglini C, Bottaro M, Dennehy C, et al. The effects of an individualized exercise intervention on body composition in breast cancer patients undergoing treatment. *Sao Paulo Med J* 2007;125(1):22-8. [published Online First: 2007/05/17]
82. Jones LW, Liang Y, Pituskin EN, et al. Effect of exercise training on peak oxygen consumption in patients with cancer: a meta-analysis. *Oncologist* 2011;16(1):112-20. doi: 10.1634/theoncologist.2010-0197 [published Online First: 2011/01/08]
83. Persoon S, Chin AMJM, Buffart LM, et al. Randomized controlled trial on the effects of a supervised high intensity exercise program in patients with a hematologic malignancy treated with autologous stem cell transplantation: Results from the EXIST study. *PLoS One* 2017;12(7):e0181313.
84. MacKinnon DP, Fairchild AJ, Fritz MS. Mediation analysis. *Annual review of psychology* 2007;58:593-614. doi: 10.1146/annurev.psych.58.110405.085542 [published Online First: 2006/09/14]
85. Process evaluation for public health interventions and research. San Francisco, CA, US: Jossey-Bass 2002.

For peer review only

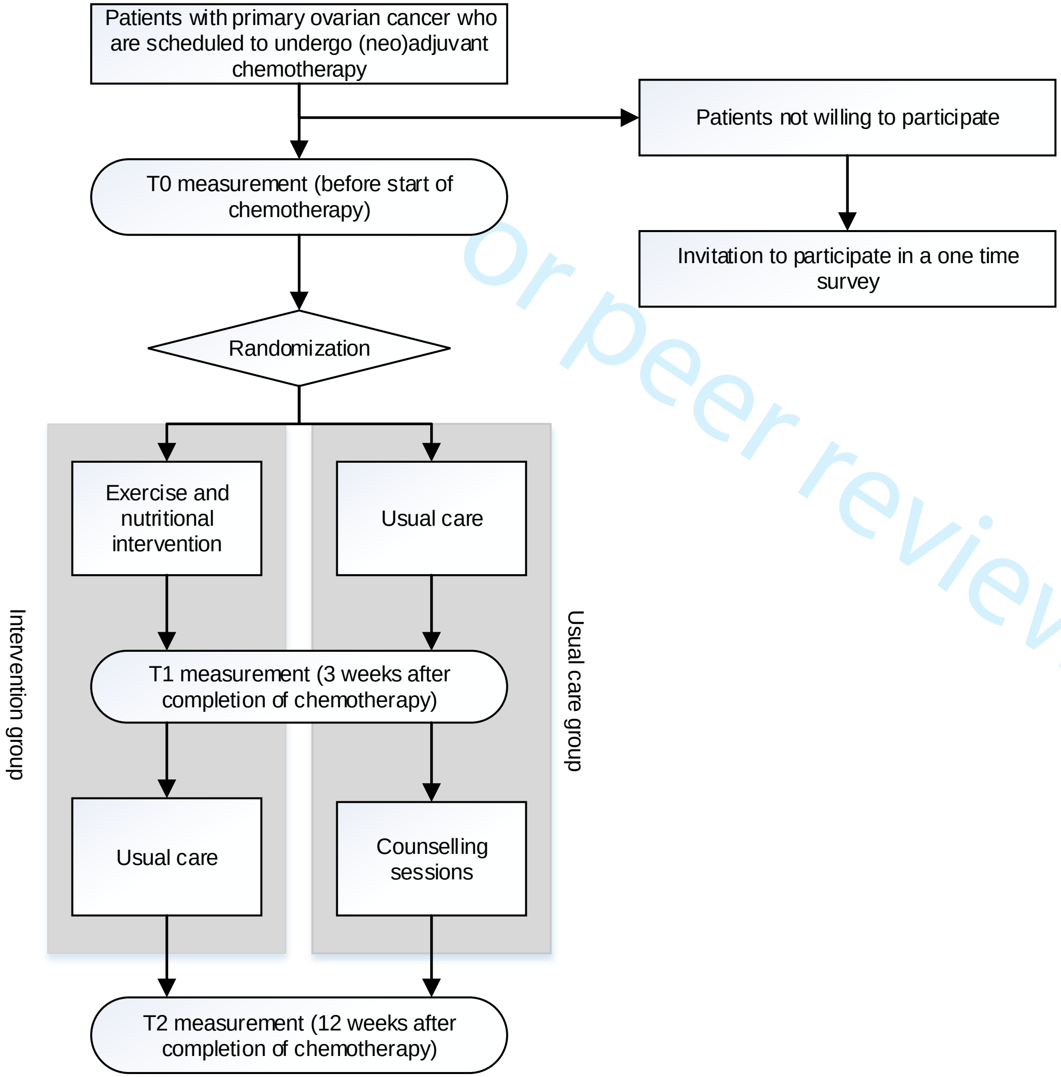

For peer review only

Supplementary table A: Overview of Behavior Change Techniques (BCT's) used to promote health behavior change in the PADOVA dietary intervention

Theory	Construct	BCT[1]	Description of BCT
Social Cognitive Theory			
	Self-efficacy	Graded tasks	To promote self-efficacy, the dietitian will stimulate the participant to set easy to perform and achievable individual goals, and will promote gradually making individual goals more difficult until the recommendation is met.
	Outcome expectations	Comparative imagining of future outcomes	The dietitian will prompt or advise the imagining and comparing of future outcomes of changed versus unchanged behavior.
		Vicarious consequences	The dietitian will prompt observation of the consequences (including rewards and punishments) for others when they perform the behavior.
	Goal setting	Goal setting (outcomes of) behavior	Individual goals with regard to dietary intake/weight will be set by the participant in consultation with the dietitian.
	Sociostructural factors	Problem solving	The dietitian and patient discuss factors that could influence achieving each goal, as well as strategies to overcome possible barriers and/or strategies to increase facilitators to achieving each goal.
		Social support (practical)	The dietitian gives advice on finding social support (e.g. practical help from family or friends) for behavior change in order to reach individual goals.
		Habit formation	The dietitian will advise on rehearsal and repetition of the behavior in the same context repeatedly so that the context elicits the behavior.
		Avoidance/reducing exposure to cues for the behavior	The dietitian will advise on how to avoid exposure to specific social and contextual/physical cues for the behavior, including changing daily/weekly routines.
		Restructuring the physical environment	The dietitian will facilitate change or advise to change the physical environment in order to facilitate performance of the wanted behavior or create barriers to the unwanted behavior.
		Restructuring the social environment	The dietitian will facilitate change or advise to change the social environment in order to facilitate performance of the wanted behavior or create barriers to the unwanted behavior.
		Information about antecedents	The dietitian will provide information about antecedents (social, environmental situations or events, emotions, cognitions) that reliably predict performance of the behavior.
		Self-reward	The dietitian will prompt self-praise or self-reward if and only if there has been effort and/or progress in performing the behavior.
		Reduce negative emotions	The dietitian will advise on ways of reducing negative emotions to facilitate performance of the behavior (includes stress-management).
Motivational interviewing			
		Pros and cons	The dietitian will advise to identify and compare reasons for wanting (pros) and not wanting (cons) to change the behavior (includes decisional balance).
		Comparative imagining of future outcomes	The dietitian will prompt or advise the imagining and comparing of future outcomes of changed versus unchanged behavior.
		Social support (unspecified)	The dietitian will advise on and how to arrange social support (e.g., from friends, family, buddies) or non-contingent praise or reward for performance of the behavior. Includes encouragement and counselling when directed at the behavior.

For peer review only

Other			
		Credible source	Dietitian from hospital provides counselling.
		Feedback on behavior	The participant will receive feedback from the dietitian on diet quality, and on the extent to which they meet the World Cancer Research Fund (WCRF)/American Institute for Cancer Research (AICR) [2] recommendations and the protein-goals [3,4].
		Feedback on outcomes behavior	The participant will receive feedback from the dietitian on their weight, Body Mass Index, body composition.
		Information about health consequences	The dietitian will inform the participant about the influence of lifestyle-related factors on the occurrence of cancer and about the potential positive effects of increased physical activity and a healthy diet (including the effect of habitual protein consumption during exercise) throughout chemotherapy.
		Instruction on how to perform the behavior	The participant will receive information from the dietitian on the WCRF/AICR recommendations (leaflet).
		Adding objects to the environment; self-monitoring of behavior; self- monitoring of outcomes of behavior	The participant will receive a self-monitoring log from the dietitian in which they can log their weight and diet. They are encouraged to weekly log their weight, and to daily log their dietary intake, with flexibility to meet individual needs and preferences.
		Action planning	An action plan for each individual goal will be discussed by the participant and dietitian.
		Discrepancy between current behavior and goal	The dietitian will point out potential discrepancies between patients' current behavior and each goal during each subsequent session.
		Review behavior goals/review outcome goals	The self-monitoring logs will be discussed with the oncology dietitian during every counselling visit to be able to monitor progress. Each goal will be reviewed and may be modified if necessary. Also, new goals may be set.
		Social reward (positive reinforcement)	The dietitian will congratulate the patient in case of success.
		Verbal persuasion about capability	The dietitian will tell the person that they can successfully perform the wanted behavior, arguing against self-doubts and asserting that they can and will succeed.

For peer review only

REFERENCES

1. Michie S, Richardson M, Johnston M, et al. The behavior change technique taxonomy (v1) of 93 hierarchically clustered techniques: building an international consensus for the reporting of behavior change interventions. *Ann Behav Med* 2013;46(1):81-95. doi: 10.1007/s12160-013-9486-6 [published Online First: 2013/03/21]
2. World Cancer Research Fund / American Institute for Cancer Research. Diet, Nutrition, Physical Activity and Cancer: a Global Perspective. Continuous Update Project Expert report, 2018.
3. Nitenberg G, Raynard B. Nutritional support of the cancer patient: issues and dilemmas. *Crit Rev Oncol Hematol* 2000;34(3):137-68. [published Online First: 2000/06/06]
4. Arends J, Bachmann P, Baracos V, et al. ESPEN guidelines on nutrition in cancer patients. *Clin Nutr* 2016 doi: 10.1016/j.clnu.2016.07.015 [published Online First: 2016/09/18]

For peer review only

Supplementary table B: Overview of the content of the PADOVA dietary counselling sessions

Counselling session	Content
First counselling session	- Introduction of dietitian and aim of dietary counselling sessions<br>- Anthropometric measures<br>o Current weight and weight history<br>o Height<br>o Body Mass Index<br>o Body composition<br>- Dietary assessment<br>o Nutrition-related illnesses or symptoms (e.g. reduced appetite, nausea, vomiting, gastrointestinal problems, chewing or swallowing difficulties)<br>o Relevant social factors (e.g. social support)<br>o Dietary analyses of current nutritional intake<br>o Current exercise and physical activity level<br>- Assessment of energy [1,2] and protein [3,4] requirements using multiple formulas<br>- Dietetic diagnosis (synthesized information from anthropometric measures and dietary assessment)<br>- Provide feedback on patients' weight, body composition and dietary intake<br>- Providing information about the influence of lifestyle and body weight related factors on the occurrence of cancer and about the potential positive effects of increased physical activity and a healthy diet (including the effects of habitual protein consumption during exercise) throughout chemotherapy.<br>- Set individual goals and action plans to achieve goals (depending on current nutritional status)<br>- Discussion of factors that could influence achieving each goal, as well as strategies to overcome possible barriers and/or strategies to increase facilitators<br>- Hand out self-monitoring logs in which patients can log their weight, dietary intake and/or physical activity.
Second – fifth counselling session	- Anthropometric measures<br>o Current weight and Body Mass Index<br>o Body composition (every other counselling session)<br>- Dietary assessment (if changed)<br>- Assessment of energy and protein requirements (if changed)<br>- (Revision of) dietetic diagnosis<br>- Discussion of filled in self-monitoring logs<br>- Discussion of potential discrepancies between current behavior and each goal<br>- Review and if necessary modification of goals and action plans
Last counselling session	- Same content as second to fifth counselling session<br>- Discussion and encouragement of self-regulation strategies to be able to maintain adherence to the World Cancer Research Fund/American Institute for Cancer Research recommendations after the end of the intervention [5]

REFERENCES

For peer review only

1. Roza AM, Shizgal HM. The Harris Benedict equation reevaluated: resting energy requirements and the body cell mass. *Am J Clin Nutr* 1984;40(1):168-82. doi: 10.1093/ajcn/40.1.168 [published Online First: 1984/07/01]
2. Energy and protein requirements. Report of a joint FAO/WHO/UNU Expert Consultation. *World Health Organ Tech Rep Ser* 1985;724:1-206. [published Online First: 1985/01/01]
3. Nitenberg G, Raynard B. Nutritional support of the cancer patient: issues and dilemmas. *Crit Rev Oncol Hematol* 2000;34(3):137-68. [published Online First: 2000/06/06]
4. Arends J, Bachmann P, Baracos V, et al. ESPEN guidelines on nutrition in cancer patients. *Clin Nutr* 2016 doi: 10.1016/j.clnu.2016.07.015 [published Online First: 2016/09/18]
5. World Cancer Research Fund / American Institute for Cancer Research. Diet, Nutrition, Physical Activity and Cancer: a Global Perspective. Continuous Update Project Expert report, 2018.

For peer review only

STANDARD PROTOCOL ITEMS: RECOMMENDATIONS FOR INTERVENTIONAL TRIALS

SPIRIT 2013 Checklist: Recommended items to address in a clinical trial protocol and related documents*

Section/item	Item No	Description	Addressed on page number
**Administrative information**			
Title	1	Descriptive title identifying the study design, population, interventions, and, if applicable, trial acronym	1
Trial registration	2a	Trial identifier and registry name. If not yet registered, name of intended registry	3
	2b	All items from the World Health Organization Trial Registration Data Set	N/A
Protocol version	3	Date and version identifier	In MEC protocol
Funding	4	Sources and types of financial, material, and other support	18
Roles and responsibilities	5a	Names, affiliations, and roles of protocol contributors	1, 18
	5b	Name and contact information for the trial sponsor	1
	5c	Role of study sponsor and funders, if any, in study design; collection, management, analysis, and interpretation of data; writing of the report; and the decision to submit the report for publication, including whether they will have ultimate authority over any of these activities	18
	5d	Composition, roles, and responsibilities of the coordinating centre, steering committee, endpoint adjudication committee, data management team, and other individuals or groups overseeing the trial, if applicable (see Item 21a for data monitoring committee)	N/A

For peer review only

Introduction

Background and rationale	6a	Description of research question and justification for undertaking the trial, including summary of relevant studies (published and unpublished) examining benefits and harms for each intervention	4,5
	6b	Explanation for choice of comparators	4,5
Objectives	7	Specific objectives or hypotheses	4,5
Trial design	8	Description of trial design including type of trial (eg, parallel group, crossover, factorial, single group), allocation ratio, and framework (eg, superiority, equivalence, noninferiority, exploratory)	5,6

Methods: Participants, interventions, and outcomes

Study setting	9	Description of study settings (eg, community clinic, academic hospital) and list of countries where data will be collected. Reference to where list of study sites can be obtained	6
Eligibility criteria	10	Inclusion and exclusion criteria for participants. If applicable, eligibility criteria for study centres and individuals who will perform the interventions (eg, surgeons, psychotherapists)	6
Interventions	11a	Interventions for each group with sufficient detail to allow replication, including how and when they will be administered	6-13
	11b	Criteria for discontinuing or modifying allocated interventions for a given trial participant (eg, drug dose change in response to harms, participant request, or improving/worsening disease)	6-13
	11c	Strategies to improve adherence to intervention protocols, and any procedures for monitoring adherence (eg, drug tablet return, laboratory tests)	6-15
	11d	Relevant concomitant care and interventions that are permitted or prohibited during the trial	N/A
Outcomes	12	Primary, secondary, and other outcomes, including the specific measurement variable (eg, systolic blood pressure), analysis metric (eg, change from baseline, final value, time to event), method of aggregation (eg, median, proportion), and time point for each outcome. Explanation of the clinical relevance of chosen efficacy and harm outcomes is strongly recommended	13-16
Participant timeline	13	Time schedule of enrolment, interventions (including any run-ins and washouts), assessments, and visits for participants. A schematic diagram is highly recommended (see Figure)	6-15, figure 1, table 3 and 4

For peer review only - http://bmjopen.bmj.com/site/about/guidelines.xhtml

Sample size	14	Estimated number of participants needed to achieve study objectives and how it was determined, including clinical and statistical assumptions supporting any sample size calculations	16
Recruitment	15	Strategies for achieving adequate participant enrolment to reach target sample size	In MEC protocol
**Methods: Assignment of interventions (for controlled trials)**			
Allocation:			
Sequence generation	16a	Method of generating the allocation sequence (eg, computer-generated random numbers), and list of any factors for stratification. To reduce predictability of a random sequence, details of any planned restriction (eg, blocking) should be provided in a separate document that is unavailable to those who enrol participants or assign interventions	6
Allocation concealment mechanism	16b	Mechanism of implementing the allocation sequence (eg, central telephone; sequentially numbered, opaque, sealed envelopes), describing any steps to conceal the sequence until interventions are assigned	6
Implementation	16c	Who will generate the allocation sequence, who will enrol participants, and who will assign participants to interventions	6
Blinding (masking)	17a	Who will be blinded after assignment to interventions (eg, trial participants, care providers, outcome assessors, data analysts), and how	13
	17b	If blinded, circumstances under which unblinding is permissible, and procedure for revealing a participant's allocated intervention during the trial	N/A
**Methods: Data collection, management, and analysis**			
Data collection methods	18a	Plans for assessment and collection of outcome, baseline, and other trial data, including any related processes to promote data quality (eg, duplicate measurements, training of assessors) and a description of study instruments (eg, questionnaires, laboratory tests) along with their reliability and validity, if known. Reference to where data collection forms can be found, if not in the protocol	13-16
	18b	Plans to promote participant retention and complete follow-up, including list of any outcome data to be collected for participants who discontinue or deviate from intervention protocols	13-16

Data management	19	Plans for data entry, coding, security, and storage, including any related processes to promote data quality (eg, double data entry; range checks for data values). Reference to where details of data management procedures can be found, if not in the protocol	16-17
Statistical methods	20a	Statistical methods for analysing primary and secondary outcomes. Reference to where other details of the statistical analysis plan can be found, if not in the protocol	16-17
	20b	Methods for any additional analyses (eg, subgroup and adjusted analyses)	16-17
	20c	Definition of analysis population relating to protocol non-adherence (eg, as randomised analysis), and any statistical methods to handle missing data (eg, multiple imputation)	16-17
**Methods: Monitoring**			
Data monitoring	21a	Composition of data monitoring committee (DMC); summary of its role and reporting structure; statement of whether it is independent from the sponsor and competing interests; and reference to where further details about its charter can be found, if not in the protocol. Alternatively, an explanation of why a DMC is not needed	Low risk study, therefore DMC is not needed
	21b	Description of any interim analyses and stopping guidelines, including who will have access to these interim results and make the final decision to terminate the trial	N/A
Harms	22	Plans for collecting, assessing, reporting, and managing solicited and spontaneously reported adverse events and other unintended effects of trial interventions or trial conduct	N/A
Auditing	23	Frequency and procedures for auditing trial conduct, if any, and whether the process will be independent from investigators and the sponsor	N/A
**Ethics and dissemination**			
Research ethics approval	24	Plans for seeking research ethics committee/institutional review board (REC/IRB) approval	5-6
Protocol amendments	25	Plans for communicating important protocol modifications (eg, changes to eligibility criteria, outcomes, analyses) to relevant parties (eg, investigators, REC/IRBs, trial participants, trial registries, journals, regulators)	In MEC protocol

For peer review only - http://bmjopen.bmj.com/site/about/guidelines.xhtml

Consent or assent	26a	Who will obtain informed consent or assent from potential trial participants or authorised surrogates, and how (see Item 32)	In MEC protocol
	26b	Additional consent provisions for collection and use of participant data and biological specimens in ancillary studies, if applicable	N/A
Confidentiality	27	How personal information about potential and enrolled participants will be collected, shared, and maintained in order to protect confidentiality before, during, and after the trial	In MEC protocol
Declaration of interests	28	Financial and other competing interests for principal investigators for the overall trial and each study site	18
Access to data	29	Statement of who will have access to the final trial dataset, and disclosure of contractual agreements that limit such access for investigators	In MEC protocol
Ancillary and post-trial care	30	Provisions, if any, for ancillary and post-trial care, and for compensation to those who suffer harm from trial participation	In MEC protocol
Dissemination policy	31a	Plans for investigators and sponsor to communicate trial results to participants, healthcare professionals, the public, and other relevant groups (eg, via publication, reporting in results databases, or other data sharing arrangements), including any publication restrictions	In MEC protocol
	31b	Authorship eligibility guidelines and any intended use of professional writers	N/A
	31c	Plans, if any, for granting public access to the full protocol, participant-level dataset, and statistical code	N/A
**Appendices**			
Informed consent materials	32	Model consent form and other related documentation given to participants and authorised surrogates	See supplementary files C & D
Biological specimens	33	Plans for collection, laboratory evaluation, and storage of biological specimens for genetic or molecular analysis in the current trial and for future use in ancillary studies, if applicable	N/A

*It is strongly recommended that this checklist be read in conjunction with the SPIRIT 2013 Explanation & Elaboration for important clarification on the items. Amendments to the protocol should be tracked and dated. The SPIRIT checklist is copyrighted by the SPIRIT Group under the Creative Commons "Attribution-NonCommercial-NoDerivs 3.0 Unported" license.

For peer review only - http://bmjopen.bmj.com/site/about/guidelines.xhtml

For peer review only

For peer review only - http://bmjopen.bmj.com/site/about/guidelines.xhtml
